# A general framework for optimising cost-effectiveness of pandemic response under partial intervention measures

**DOI:** 10.1038/s41598-022-23668-x

**Published:** 2022-11-14

**Authors:** Quang Dang Nguyen, Mikhail Prokopenko

**Affiliations:** Centre for Complex Systems, Faculty of Engineering, University of Sydney, Darlington, NSW 2008 Australia

**Keywords:** Infectious diseases, Computer science

## Abstract

The COVID-19 pandemic created enormous public health and socioeconomic challenges. The health effects of vaccination and non-pharmaceutical interventions (NPIs) were often contrasted with significant social and economic costs. We describe a general framework aimed to derive adaptive cost-effective interventions, adequate for both recent and emerging pandemic threats. We also quantify the net health benefits and propose a reinforcement learning approach to optimise adaptive NPIs. The approach utilises an agent-based model simulating pandemic responses in Australia, and accounts for a heterogeneous population with variable levels of compliance fluctuating over time and across individuals. Our analysis shows that a significant net health benefit may be attained by adaptive NPIs formed by partial social distancing measures, coupled with moderate levels of the society’s willingness to pay for health gains (health losses averted). We demonstrate that a socially acceptable balance between health effects and incurred economic costs is achievable over a long term, despite possible early setbacks.

## Introduction

The COVID-19 pandemic has generated enormous health, economic and social costs, causing a significant loss of life, adversely affecting the population health, and creating a substantial shock to national and global economies. The pandemic dramatically reduced life expectancy and increased premature mortality^1–3^, often impairing the capacity of healthcare systems to deal with the crisis^4,5^. In parallel, various supply chains, labour and equity markets worldwide, and entire sectors of economy, such as tourism, energy and finance sectors, have also suffered very substantial and cascading impacts^6–9^. These non-trivial challenges created a major need for appropriate and sustainable pandemic responses capable of balancing both health and socioeconomic consequences: a problem which continues to evade simple solutions. On the one hand, it proved to be difficult to objectively model and quantify the health and economic costs in comparative terms^10,11^. On the other hand, contrasting the health and socioeconomic impacts was often dependent on fairly subjective perspectives of policy- and decision-makers^12–14^, as well as cultural differences, political influences, and other factors affecting trust in governments and health authorities^15,16^. Typically, attempts to “optimise” multiple objectives were carried out under severe pressure, failing to flatten epidemic curves and prevent an economic downturn. Consequently, society-wide responses were prone to conflicting influences, including (geo-)political, economic, social and behavioural factors, and produced sub-optimal or ill-timed intervention measures.

Initial responses to the unfolding pandemic, dominated by an abundance of caution and employing mostly non-pharmaceutical interventions (NPIs), have varied in their scope and effectiveness^17–21^. Once vaccines became broadly available, the intervention focus has changed to mass vaccination campaigns which have also shown a varying degree of success, reducing the pandemic impact and allowing many affected societies and economies to moderately recover^22–25^. Nevertheless, subsequent pandemic waves generated by emerging viral variants of concern, such as the Delta and Omicron variants of SARS-CoV-2, continued to severely disrupt individual lives and national economies^26–28^. It is likely that the ongoing spread and evolution of the SARS-CoV-2 virus will continue to affect the recovery efforts, with periods of relative calm interleaved with renewed outbreaks and pandemic waves. Possible oscillatory pandemic dynamics can be expected even after a transition to endemicity, with only transient herd immunity developing in the near to mid-term, without long-lasting transmission-blocking immunity^29^. Moreso, a transition to endemicity is dependent on the interplay of human behaviour, demographics, susceptibility, immunity, and emergence of new viral variants^30^.

It is increasingly evident that maintaining a strict intervention policy is hardly possible over a long time, especially if such a policy involves persistent social distancing and stay-at-home orders. Given the imperative to balance population health against unacceptable or severe socio-economic impacts, in presence of emerging outbreaks, there is a clear need for a more refined approach centred on adaptive, contextual and cost-effective interventions. A successful approach should not only offer a way to reconcile health and socio-economic perspective, but also utilise an unbiased methodology for a search of optimal or near optimal policies, non influenced by modelling or policy-making preferences.

This study addresses several of these challenges. Firstly, we quantify the cost-effectiveness of intervention measures using the Net Health Benefit (NHB) approach which balances *both health and economic costs*. In doing so, we formulate a search space for suitable interventions in terms of two thresholds. The first one is the “willingness to pay” (WTP) defined as an acceptable threshold that can be paid for an additional unit of health benefit, such as the disability-adjusted life years (DALY) averted. The second threshold is the level of maximal compliance with the social distancing (SD) measures that may be imposed on the population. Varying the WTP per DALY averted for different SD levels allows us to systematically explore the policy search space with respect to the resultant health benefits.

Secondly, in modelling the transmission and control of the COVID-19 pandemic we adopt an agent-based model (ABM) approach, described in "Methods" and Supplementary Material: Agent-based Model. Each individual is represented by a computational agent stochastically generated based on relevant demographic (census) data. These agents are simulated over time with respect to their interactions in different social contexts (e.g., residential, workplace, educational), probabilistically transmitting infection, getting ill, recovering from the disease, and so on^19,31^. This high-resolution approach allows us to capture not only the *population heterogeneity*, but also the *fluctuating compliance* with social distancing that may vary (i) across the agents, and (ii) over time. In each simulated scenario, we do not assume homogeneous or persistent adherence of individuals to a fixed SD level, instead limiting the fraction of compliant individuals, dynamically selected at each time point, by a maximal level. Thus, the model can evaluate *partial* interventions shaped by complex SD behaviours fluctuating between the strongest possible commitment ($SD = SD_{max}$) and extreme fatigue ($SD = 0$), heterogeneously and dynamically distributed across the population.

Following^31^, social distancing is defined holistically, comprising various behavioural changes aimed to reduce the intensity of individual interactions during a given restriction period. These changes typically include stay-at-home restrictions, travel reduction, as well as physical distancing, mask wearing, and other measures. Dependent on the social context, each compliant agent may reduce the intensity of interactions with their household members, neighbours and coworkers/classmates. A typical scenario also assumes other NPIs, such as case isolation and home quarantine, as well as a *partial* mass-vaccination coverage affecting a proportion of the population (see "Methods" and Supplementary Material: Vaccination Strategy). In general, vaccination campaigns can be either pre-emptive (vaccination between outbreaks) or reactive (vaccination during an outbreak). We consider pre-emptive vaccination campaigns in relation to future outbreaks of the COVID-19. In other words, we assume that a vaccination campaign, covering a significant fraction of the population (e.g., 85%), is carried out before such an outbreak caused by an emerging variant of concern. Before exploring the search space of interventions, we calibrated and validated the ABM using a case study: an outbreak of the Delta variant in New South Wales, Australia, during June–November 2021 (see Supplementary Material: Model Validation).

Finally, the study utilises a reinforcement learning (RL) algorithm exploring the search space of feasible cost-effective NPIs. Rather than formulating an NPI in advance, specifying exact SD compliance levels for each time interval, the RL algorithm constructs possible interventions dynamically. This is achieved by selecting possible future SD actions based on relative success of prior simulations, i.e., using a “reward” signal. These rewards may reinforce or weaken the selection probability of the corresponding SD actions, so that better policies may emerge after a sufficiently long learning period. In order to ensure feasibility, the actions are selected at some realistic decision points (e.g., weekly). The interventions that outperform other candidates in terms of cost-effectiveness, as measured by the NHB—that is, balancing both health and economic costs—contribute to further simulations, improving the NHB over the learning process. Crucially, the RL-based approach eliminates subjectivity in selecting feasible NPIs, by removing bias towards several frequently considered preferences for “short and snap” lockdowns^32^, mandatory large-scale social distancing campaigns^17,18^, or loose restrictions in style of de-facto “herd immunity” approaches^33,34^. In principle, resultant interventions produced by the RL algorithm may have different temporal profiles unencumbered by such subjective choices, as long as the policy changes are deemed feasible and the outcomes are superior.

Our comparative analysis shows that it is possible to generate a significant net health benefit by a feasible pattern of partial social distancing measures. These temporal patterns, produced by an unbiased machine learning approach, adapt to different values of the society’s willingness to pay for a single lost (disability-adjusted) year of life. A resultant profile typically starts with near-maximal levels of adherence to social distancing ($SD \approx SD_{max}$) and allows for a significant relaxation of social distancing to much lower levels ($SD \lessapprox 0.2$) after several weeks. While the period of higher commitment is dependent on the WTP threshold, this dependence does not preclude viable SD interventions progressing even for relatively low values of the threshold $SD_{max}$. However, with higher $SD_{max}$ thresholds, the relaxation of strict compliance measures becomes viable sooner. Interestingly, the net health benefit produced by mid-level compliance with NPIs carried out under a moderate willingness-to-pay setting is commensurate with the benefits yielded by higher $SD_{max}$ and WTP.

In adaptive strategies, the level of SD imposed on the population in a given week changes in response to the current pandemic state, and thus depends on previous actions in context of the accepted WTP. Importantly, adaptive NPIs outperform possible alternatives, including fixed, random or zero social distancing, across the entire range of considered WTP and $SD_{max}$ thresholds. We demonstrate that the higher cumulative NHB attained by adaptive NPIs non-linearly balances the incurred economic costs and sustained health losses, achieving longer-term advantages despite possible early setbacks. Overall, these findings suggest that the choice between “health” and “economy” is a false choice, and an adaptive policy may achieve a socially acceptable balance even under significant constraints.

## Results

Using our agent-based model and reinforcement learning algorithm, we investigated three settings of maximal compliance with social distancing ($$SD_{max} = 0.3$$, $$SD_{max} = 0.5$$, and $$SD_{max} = 0.7$$, in other words, the maximal fraction of compliant population set at 30%, 50% and 70% respectively). For each of these levels, we varied the “willingness to pay” (WTP) across three thresholds: $10K per DALY, $50K per DALY and $100K per DALY. Each (SD, WTP) setting was evaluated in terms of the Net Health Benefit (NHB), generated by the adaptive NPIs learned by the RL algorithm over 19 simulated weeks of the disease spread, and further analysed with respect to two separate NHB components: the economic costs and health effects. Each simulation run was carried out for a typical pandemic scenario developing in an Australian town (see "Methods" and Supplementary Material: Agent-based Model), with the economic costs adjusted in proportion to the population size, and DALY losses computed from the estimates of incidence and fatalities generated by multiple ABM simulations.

### Dynamics of adaptive NPIs

**Figure 1:**
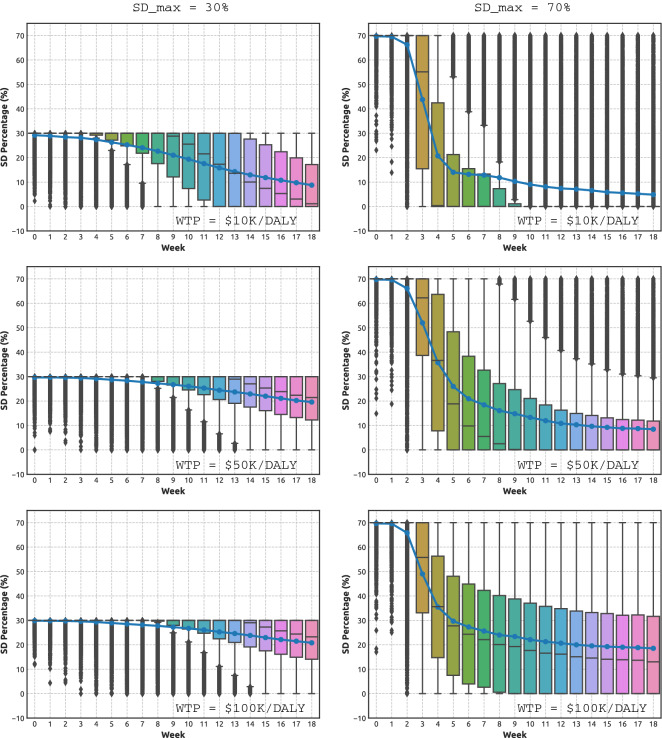
Adaptive NPIs, learned under different combinations of maximal SD levels $$SD_{max}$$ and WTP, over more than 14,000 simulations. Left: $$SD_{max} = 0.3$$. Right: $$SD_{max} = 0.7$$. Top: WTP is set at $10K per DALY. Middle: WTP is set at $50K per DALY. Bottom: WTP is set at $100K per DALY. Boxplots show the distribution of data over the quartiles, with box body capturing the mid-50% of the distribution. The curves shown with blue colour trace the mean values of the SD levels attained in each week. Outliers are shown in black.

Figure 1 contrasts two $SD_{max}$ settings, 30% and 70%, across three considered WTP levels, tracing the level of compliance with social distancing over time. A similar comparison between $SD_{max}$ set at 50% and 70% is presented in Supplementary Material (Fig. 14). All adaptive NPIs, triggered when the number of detected infections exceeds a threshold (invariably, during the first week), begin at the maximal considered SD level, i.e., $SD \approx SD_{max}$. In general, the SD level reduces over time. While this reduction is gradual and almost linear for the low-commitment setting, $SD_{max} = 0.3$ (Fig. 1: left panels), it is more abrupt and non-linear for the high-commitment setting, $SD_{max} = 0.7$, with the decline evident after just a few weeks (Fig. 1: right panels). In other words, higher initial levels of SD compliance allow for NPIs with shorter periods of stricter stay-at-home orders.

The differences across the patterns produced by distinct $$SD_{max}$$ settings strongly suggests that an early suppression of outbreaks helps to increase the NHB: an outcome achieved even with SD levels declining over time. Importantly, a comparison of the top, middle and bottom panels of Fig. 1 shows that the SD levels attained at the end of simulations depend on the WTP threshold, with the higher WTP values generating higher convergent SD commitments across all considered levels of $$SD_{max}$$. For example, for WTP set at $10K per DALY, the NPIs beginning at $$SD_{max} = 0.7$$ (top-right panel) converge to merely 5% of SD compliance, while for WTP set at $100K per DALY, the NPIs beginning at $$SD_{max} = 0.7$$ (bottom-right panel) remain above 20% of SD compliance at the end. A similar tendency is observed for low maximal commitment $$SD_{max} = 0.3$$ as well, with convergent SD levels differentiated between 10% of SD compliance (top-left panel) and slightly below 20% of SD compliance (bottom-left panel). This highlights the role of WTP threshold in reducing the DALY losses while minimising the corresponding economic costs.

The reported observations are robust, as indicated by boxplots shown in Fig. 1, with the majority (at least 50% shown within the box body) of the simulations following the described patterns.

### Dynamics of net health benefit

**Figure 2:**
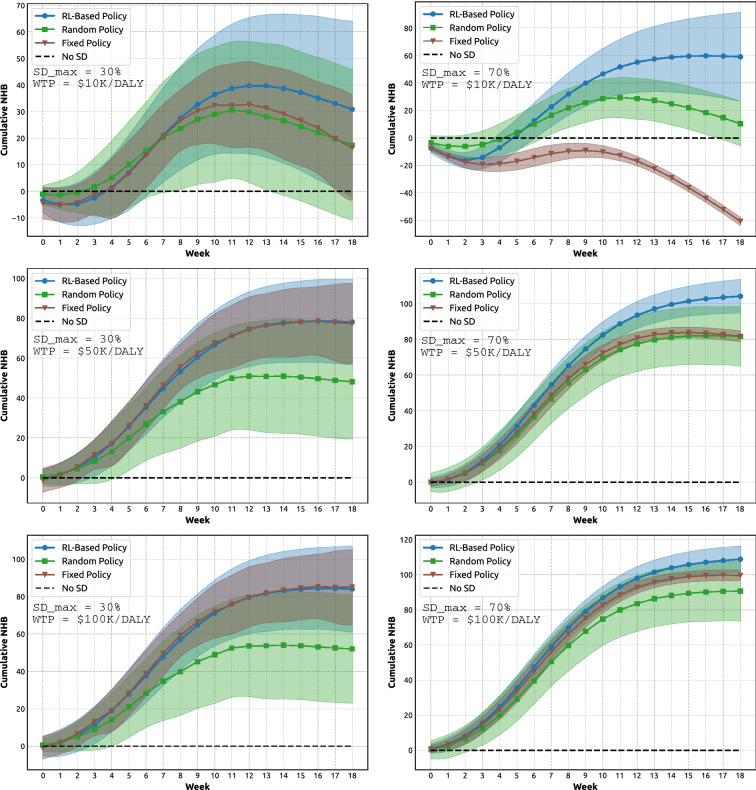
A comparison of cumulative Net health benefit (NHB) generated by the adaptive NPIs, fixed SD NPIs, random SD policies, and zero SD policies. Left: $$SD_{max} = 0.3$$. Right: $$SD_{max} = 0.7$$. Top: WTP is set at $10K per DALY. Middle: WTP is set at $50K per DALY. Bottom: WTP is set at $100K per DALY. Shaded areas show standard deviation.

Having examined dynamics of the adaptive NPIs produced by reinforcement learning, we turn our attention to dynamics of the corresponding NHB generated by these NPIs. Figure 2 compares adaptive NPIs against several competing intervention approaches, across the considered threshold levels of $SD_{max}$ and WTP (see also Supplementary Material, Fig. 15). The alternatives include: (i) fixed SD levels, when SD is set at $SD_{max}$ for the entire duration of the simulation; (ii) random SD levels, randomly fluctuating between 0 and $SD_{max}$ during the simulation; and (iii) no social distancing (zero SD). The adaptive NPIs outperform all the contenders, achieving a higher cumulative NHB for the majority of (SD, WTP) settings, and the same NHB for two settings.

Importantly, dynamics of the NHB over time are non-linear, allowing us to contrast short-term and long-term advantages of the adopted measures. The adaptive NPIs generate a superior cumulative NHB despite some initial losses or slow gains. In particular, the low WTP threshold of $10K per DALY, traced in the top panels of Fig. 2, produces negative NHB for a number of weeks: three weeks for $$SD_{max} = 0.3$$ and four weeks for $$SD_{max} = 0.7$$, followed by positive cumulative NHB during the rest of simulation. For higher WTP thresholds, $50K per DALY and $100K per DALY (shown in the middle and bottom panels), the NHB remains positive during an initial period, growing relatively slowly, followed by a more rapid NHB increase plateauing towards the end.

In some cases, during the initial period (i.e., the first few weeks), the cumulative NHB produced by the adaptive interventions is smaller than the NHB produced by the alternatives, but this is invariably replaced by higher NHB gains generated by adaptive interventions over a longer term. Thus, the intervention window offered by the initial period is crucial for an effective control of the pandemic, regardless of the society’s willingness to pay per DALY loss averted. This observation aligns with the other studies advocating early SD measures aimed to prevent escalation of outbreaks^35–37^, indicating that early short-term sacrifices yield longer-term benefits. The balance between short- and long-term advantages is most striking for the low WTP threshold of $10K per DALY, while the higher WTP thresholds extract the NHB gains almost immediately.

The approach with zero SD intervention is obviously not a serious contender, but offers a useful baseline in terms of delineating these short- and long-term advantages. Specifically, the time point when the cumulative NHB of an adaptive NPI exceeds the corresponding level for the zero SD intervention marks the point when the adaptive intervention starts to generate a longer-term benefit.

A fixed-SD intervention presents stronger competition, and achieves the same NHB as the adaptive policy in two cases: low maximal SD commitment ($$SD_{max} = 0.3$$) for middle and high WTP thresholds: $50K per DALY and $100K per DALY (shown in middle-left and bottom-left panels). These two outcomes suggest that when the the society’s willingness to pay per DALY averted is high, the low maximal SD commitment, such as $$SD_{max} = 0.3$$, constraints the scope for adaptive interventions. Nevertheless, even under this constraint, the proposed reinforcement learning approach is successful in finding adequate interventions which are as effective as their fixed SD counterparts while being less stressful for the society.

Continuing with Fig. 2, we also point out that the fixed SD approach essentially fails in some settings (e.g., high maximal SD commitment, $$SD_{max} = 0.7$$, for low WTP threshold: $10K per DALY), and sometimes, performs as poorly as the random SD approach (e.g., high maximal SD commitment, $$SD_{max} = 0.7$$, for middle WTP threshold: $50K per DALY). The lower WTP thresholds bias the NHB to weigh the economic costs of intervention higher than the health effects (averted DALY losses). Evidently, the fixed SD intervention cannot cope well with this bias, especially when there is a scope for higher compliance with the stay-at-home restrictions. In contrast, the adaptive NPIs perform very well under the higher maximal SD compliance, such as $$SD_{max} = 0.7$$, extracting a higher NHB than the alternatives, with the relative gains being much higher for the smallest considered WTP threshold.

Finally, a comparison between the left and right panels of Fig. 2 shows that the higher levels of $SD_{max}$ allow the interventions to attain the higher NHB gains, regardless of the WTP threshold, and this potential is fully realised by the adaptive NPIs.

**Figure 3:**
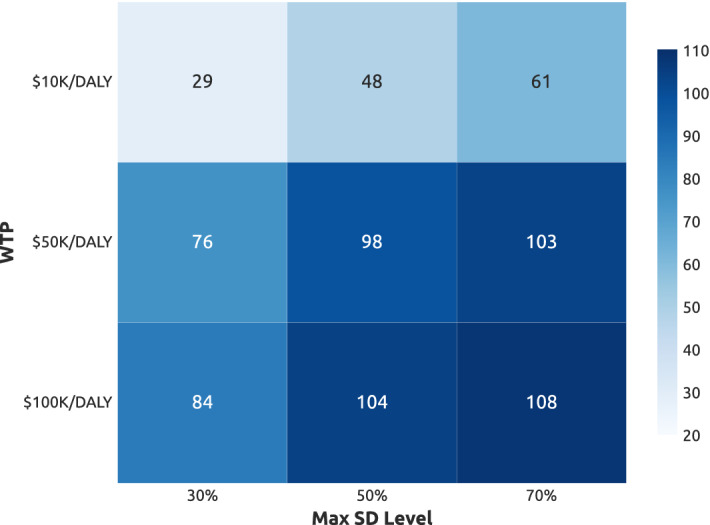
Cumulative Net Health Benefit (NHB) generated by adaptive NPIs, evaluated in the space of two threshold parameters: maximal SD level $SD_{max}$ and willingness-to-pay (WTP) threshold. NHB values shown in each cell are the mean values produced over approximately 14,000 simulations.

Figure 3 summaries the average cumulative NHB generated by adaptive NPIs trained under different configurations of the WTP thresholds and the maximal SD compliance levels $SD_{max}$. The heatmap exhibits a clear NHB gradient towards the higher WTP and $SD_{max}$. However, as $SD_{max}$ increases (left to right), the relative NHB gains diminish. Hence, stricter intervention measures offer a relatively smaller gains in the health benefit, especially for higher WTP thresholds. Similarly, as WTP threshold increases (top to bottom), there are marginal gains in the corresponding NHB. This indicates that a progressively higher WTP threshold does not necessarily translate into a proportionally smaller DALY losses. Arguably, a mid-level WTP threshold coupled with mid-level compliance with the stay-at-home restrictions attains the net health benefit comparable with the higher WTP and $SD_{max}$.

### Composition of the Net Health Benefit (NHB)

**Figure 4:**
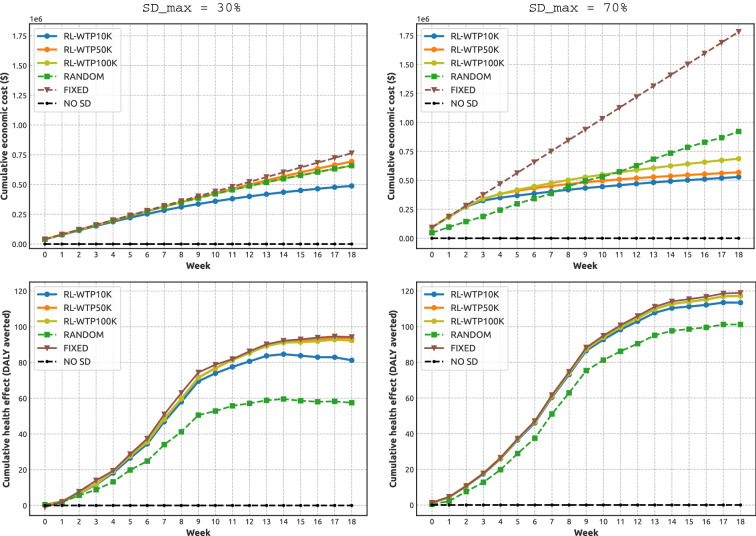
Components of the Net Health Benefit (NHB): mean values of cumulative economic costs (dollars) and cumulative health effect (DALY averted), shown for different NPIs: adaptive SD with three WTP thresholds ($10K per DALY, $50K per DALY and $100K per DALY), random SD, fixed SD, and zero SD. Left: the maximal SD level $$SD_{max}$$ is set at 30%. Right: the maximal SD level $$SD_{max}$$ is set at 70%.

The NHB is shaped by two components: economic costs and health effects (i.e., averted DALY losses), and we now analyse this composition explicitly. Figure 4 compares the economic costs and health effects (averted DALY losses) accumulated over the studied period in response to different NPIs. As above, the NPIs include adaptive SD learned across three WTP thresholds ($10K per DALY, $50K per DALY and $100K per DALY), as well as three alternatives: random SD, fixed SD, and zero SD. The analysis is carried our for three maximal levels of compliance: $$SD_{max} \in \{0.3, 0.5, 0.7\}$$, see also Supplementary Material (Fig. 16).

The zero SD interventions provide a baseline, showing zero economic costs and zero averted DALY losses (i.e., substantial health losses). The fixed SD interventions provide an opposite baseline, showing linearly growing economic costs (with the slope determined by the constant costs incurred per week in order to maintain the maximal SD level), and significant cumulative health effects in terms of averted DALY losses. The economic costs of the fixed SD interventions are the highest among alternatives, allowing to generate the highest associated health effects. Random SD interventions produce the economic costs and health effects between these two opposite baselines.

The adaptive SD interventions outperform its random alternatives, producing outcomes which are closer to one of the opposite baselines, across all considered levels of $$SD_{max}$$. In particular, despite incurring lower costs than the fixed and random SD interventions, the adaptive NPIs approach the maximal health effects generated by the SD interventions fixed at $$SD_{max}$$. In other words, the economic costs of adaptive NPIs markedly slow their growth over time, reaching the levels significantly below the costs of the fixed SD interventions, while the corresponding averted DALY losses are almost as high as the DALY losses averted by the fixed SD interventions. In summary, the adaptive NPIs achieve a beneficial trade-off between the economic costs and health effects, demonstrating long-term sustainability. As expected, the NPIs derived using the lower WTP threshold ($10K per DALY) yields lower economic costs, but generates worse health effects (that is, averts fewer DALY losses). Conversely, the NPIs derived using the higher WTP threshold ($100K per DALY) leads to higher economic costs, but achieves better health effects (averts more DALY losses).

**Figure 5:**
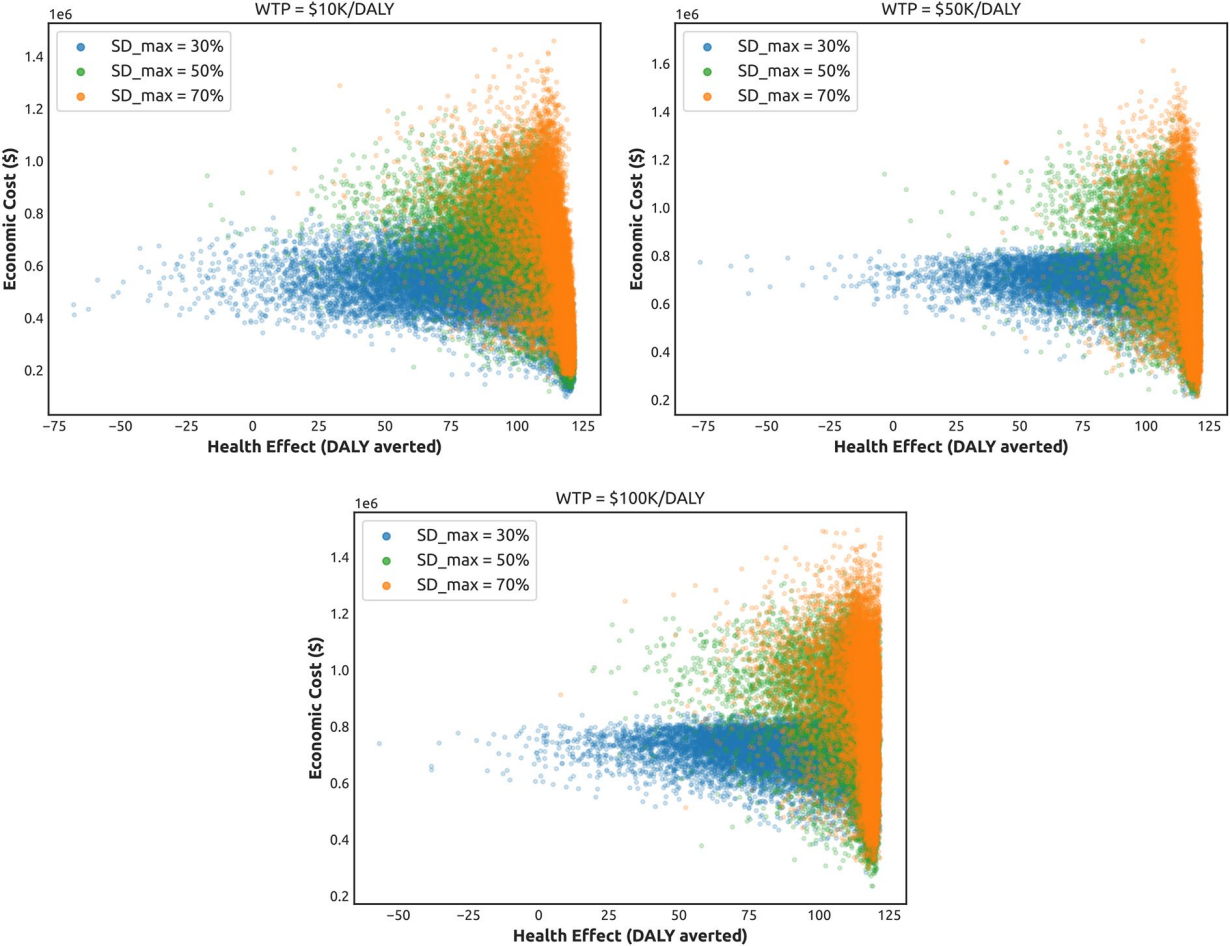
Colour plots of the Net Health Benefit (NHB) as a function of cumulative health gains (horizontal axis) and cumulative economic costs (vertical axis). The cumulative NHB is shown for more than 1000 simulations, carried out for different maximal SD levels: $$SD_{max} = 0.3$$ (blue), $$SD_{max} = 0.5$$ (green), and $$SD_{max} = 0.7$$ (orange). The colour plots are shown for adaptive NPIs with three WTP thresholds: $10K per DALY, $50K per DALY and $100K per DALY.

Finally, we consider a phase diagram of the Net Health Benefit dynamics with respect to two cumulative NHB components: the health effects in terms of DALY losses averted by adaptive NPIs and the economic costs. The diagram, shown in Fig. 5, displays results of the adaptive NPIs carried out with different thresholds for WTP and maximal SD compliance $SD_{max}$. It reveals that the search-space sampled by the *optimised* adaptive NPIs is well-structured, with the areas corresponding to different values of $SD_{max}$ clearly delineated. In particular, the area formed by NPIs operating under $SD_{max} = 0.3$ covers the region with low economic costs and low health effects. In contrast, the area formed by NPIs operating under $SD_{max} = 0.7$ covers the region with medium-to-high economic costs and relatively high health effects, but this region has a complex narrow shape, highlighting difficulties of exploring the search-space. The area formed by NPIs operating under medium maximal compliance, $SD_{max} = 0.5$, is large and well-shaped, including the region across an almost entire scale of economic costs and medium-to-high health effects. The most attractive part of all three regions (with low economic costs and high health effects) is reachable in principle but occupies a narrow tip of the attained search-space, with the search being challenged more for higher WTP thresholds.

## Discussion

Continuing efforts to control the COVID-19 pandemic typically combine mass vaccination campaigns and diverse non-pharmaceutical interventions (NPIs)^22–24,31^. In general, the socio-economic costs of the imposed NPIs are significant due to scarcity of resources, unequal wealth distribution across different socio-economic profiles, and wavering social acceptance of NPI restrictions driven by the fatigue with lockdowns and other measures. Not surprisingly, the task of finding a cost-effective allocation of finite resources generated an active research effort^38–40^. This task is likely to remain an important societal challenge, exacerbated by continuously evolving variants of the novel coronavirus which reduce the chances of completely eradicating the COVID-19 in the near future.

Traditional approaches quantifying the cost-effectiveness of interventions, such as the methods based on incremental cost-effective ratio (ICER)^41,42^, have encountered some limitations in determining and interpreting cost-effectiveness outcomes, carrying out sensitivity or scenario analysis, ranking strategies, as well as in addressing equity concerns^43,44^. In particular, it was argued that “observing change in the ICER does not necessarily imply that a strategy is more or less cost effective than in the reference-case analysis”^44,45^. More recent approaches measuring cost-effectiveness utilised the net-benefit analysis. In particular, the concept of Net Health Benefit (NHB) was introduced to formalise the cost-effectiveness problem, linearising the balance between the economic costs and health effects^46^. Formally, the NHB quantifies the difference between the health effects of two interventions: the one which is being evaluated and the baseline which is not subject to evaluation, given the corresponding cost incurred at a pre-defined cost-effectiveness threshold^47^. Similarly, the Net Monetary Benefit (NMB) was defined as a reformulation of the NHB^48^. Both NHB and NMB were shown to offer advantages in building regression-based models used in economic evaluations^43^. Over the last years, the ICER-based and the net-benefit-based methods have been used to evaluate different intervention approaches aimed to control the spread of COVID-19^38,39,49–51^.

In this work, we proposed an approach to quantify the cost-effectiveness of complex intervention measures using the NHB method accounting for both economic costs and health effects. In doing so, we simulated the COVID-19 pandemic scenarios unfolding within a representative heterogeneous population. Using an agent-based model (ABM), we compared various intervention measures, including adaptive NPIs derived by a reinforcement learning (RL) algorithm employed to maximise the NHB. The search-space for the adaptive NPIs was formed by two thresholds: (i) the “willingness to pay” (WTP) per unit of the disability-adjusted life years (DALY), and (ii) the level of maximal compliance with the social distancing measures $SD_{max}$. In choosing the three representative levels of WTP per DALY that we considered as thresholds, we followed several prior studies (See "Methods": Willingness to pay).

In recent past, several RL methods have been applied to obtain optimal NPIs in context of the COVID-19, including RL-based training of a three-level lockdown policy for a simulated population of ten thousand people^52^, which was shown to outperform age-based lockdowns^53^ and n-work-m-lockdowns^54^. An RL meta-agent-based intervention was derived as a school closure strategy in Great Britain^55^, while a multi-agent approach to optimise social distancing was evaluated across India^56^.

The approach presented in our study extends the state-of-the-art in three ways. Firstly, we demonstrate that an NHB-based approach can deliver a sustainable, adaptive, and contextual cost-effective SD intervention policy. The WTP threshold balances economic costs and health effects, contextualising the NHB given the society’s willingness to pay for DALY losses averted. Put simply, the higher the WTP, the more weight is placed on the potential health gains achieved by the incurred economic costs. Given the WTP threshold, the adaptive interventions carried out under an $$SD_{max}$$ limit are shown to be sustainable over time, reconciling both competing objectives in the long run. This indicates that these objectives do not have to be directly contrasted and various trade-offs are possible in the (WTP, SD) space. Importantly, these trade-offs are achievable for medium settings of $$SD_{max}$$, such as $$SD_{max} = 0.5$$, and WTP, e.g., WTP = $50K per DALY. This shows that future outbreaks can be controlled reasonably well even when the population response to NPIs is partial and fluctuating over time. Such partial response at a medium level of compliance may generate the NHB commensurate with the benefits of a higher but more demanding $$SD_{max}$$ limit. Crucially, these trade-offs are realised over a period of several months, and there are clear differences in the cumulative NHB generated during a short- and a long-term. Our results clearly show that a preference of “economy” over “health” does not bring long-lasting benefits, and short-term gains quickly dissipate over time. This highlights the need to take a long-term perspective in evaluating net health benefit, considering a sufficiently long response horizon.

Secondly, we capture the heterogeneity of human responses within the fluctuating fraction of compliant population. The ABM simulation not only represents the population heterogeneity, but also accounts for the stochasticity of individual choices with respect to compliance with the SD restrictions at any given time. Thus, the adaptive NPIs learned by the RL algorithm are broadly applicable to populations characterised by diverse socio-economic profiles.

Thirdly, the proposed method producing adaptive NPIs significantly reduces, and ideally removes, the subjectivity and bias which are often present in the public health decision-making. The learning algorithm considers a sufficiently long time horizon without setting any preferences or assuming particular patterns for interventions. Given the high temporal resolution of decision points (weekly) and the continuous range of the compliance levels (between zero and $SD_{max}$), the learned SD profiles are relatively unconstrained, and yet exhibit smooth trajectories, without abrupt changes. The approach also allows for a principled comparative analysis between the adaptive NPIs and their alternatives, such as fixed, random or zero SD interventions.

In our approach, the decisions about compliance are not taken by the agents. Instead, the interventions assume that a fraction of the population is compliant with the level imposed by a centralised decision-maker. At each simulated day, we assign the compliant agents differently, so that the distribution within the population varies across time. Yet the agents do not individually decide on compliance based on personal risk-benefit considerations. An important extension of this framework would include behavioural factors which are highly influenced by personal perceptions of risk^57–60^, as well as peer group pressure^61,62^ and social media messaging^63–65^. Incorporating opinion dynamics and risk-benefit analysis within an agent-based model continues to be a subject of future research.

The limitations of the study include the relatively small size of the simulated population (a typical town). The ABM itself was calibrated for a much larger population size, i.e., across millions of agents simulated at the state level (New South Wales). However, learning adaptive interventions at high temporal and SD-level resolutions demanded the reduction in the size of simulated population. In turn, we needed to proportionally reduce the economic costs incurred at the state level as a result of NPIs, assuming a linear scale. In order to make the results generalisable to larger population contexts, one would need to account for significant differences in NPI uptake and effectiveness across various populations and countries^66^. Finally, the learned adaptive interventions do not guarantee global optimality, and solutions with even higher NHB are possible in principle.

Despite these limitations, the proposed approach can be effectively used to support policy- and decision-makers. On the one hand, the simulation of various pandemic scenarios, distinct WTP and compliance thresholds, as well as different demographic profiles, can inform policy makers on the cost-effectiveness and possible trade-offs achieved by adaptive interventions, trained by the reinforcement learning method coupled with a calibrated ABM. On the other hand, the adaptive interventions may be compared against actual real-world data, so that the detected discrepancies may identify the divergence of some underlying assumptions, further elucidating the required responses and necessary adjustments. This may be particularly relevant in case of new variants of concern emerging during the anticipated endemic phase of the COVID-19. For example, the framework is directly applicable to modelling pandemic responses to the Omicron variant. Such an application would require straightforward changes in the ABM parameters, e.g., transmissibility, etc. (see Supplementary Material: Table 4), vaccination efficacies, and the clinical burden rates (recovery distribution and fatality rate, see Supplementary Material: Recovery and Fatality). This is a subject of future work. Importantly, the proposed framework can enable a comprehensive evaluation of the role played by two key thresholds (WTP and $SD_{max}$), offering insights into the interplay between individual human behaviour and the emergent social dynamics during pandemics.

## Methods

We propose a framework to search for cost-effective SD interventions balancing the health effects (averted DALY losses) and the costs associated with SD intervention measures. Decisions on SD interventions are taken with respect to a maximal SD level, determining a population fraction complying with the stay-at-home restrictions aimed to control the COVID-19 transmission in a typical Australian town. Our framework comprises three main components: (i) a method to evaluate the cost-effectiveness of SD intervention measures, (ii) an agent-based model (ABM) to simulate the effect of these interventions on the progression of the COVID-19 disease, and (iii) an RL algorithm to optimise an adaptive SD intervention simulated within the ABM and evaluated in terms of cost-effectiveness. The following sections describe these components in further detail. Our study did not involve experiments on humans/human data or the use of human tissue samples. The anonymised census data, which is related to the build of the ABM, are publicly available from the Australian Bureau of Statistics.

### Net health benefit

In order to evaluate the cost-effectiveness of NPI interventions, we quantify the net health benefit (NHB)^46,67^. The NHB captures the difference between the health effect of a new intervention and the comparative health effect, given the associated cost incurred at some pre-defined cost-effectiveness thresholds. The cost and the health effect of the new intervention are measured against the “null” intervention, that is, in presence of some baseline interventions which are not subject to evaluation^47^. In our study, the null set comprises only the base interventions, i.e., case isolation (CI), home quarantine (HQ), and travel (border control) restrictions (TR). Hence, we evaluate cost-effectiveness of the NPIs shaped by social distancing (SD), beyond that of the CI, HQ and TR interventions. The rate modulating the health effects’ comparison is called “willingness to pay” (WTP), defined as a maximum monetary threshold that the society accepts as the cost of an additional unit of health gained due to the new intervention. The NHB of the SD intervention is defined as follows:

1 $$\begin{aligned} \text {NHB} = \mu _{E_{SD}} - \frac{\mu _{C_{SD}}}{\lambda } \end{aligned}$$

where $\mu _{E_{SD}}$ is the mean of the health effect $E_{SD}$ produced by the SD intervention, $\mu _{C_{SD}}$ is the mean of the cost $C_{SD}$ incurred by this intervention, and $\lambda$ is the WTP set by policy makers or public health programs.

The corresponding health effect $E_{SD}$ of the SD intervention is computed by comparing the health losses averted by the evaluated intervention to the losses of the null intervention:

2 $$\begin{aligned} E_{SD} = L_{0} - L_{SD} \end{aligned}$$

where $L_{0}$ and $L_{SD}$ are the health losses for the null and SD interventions respectively (see Fig. 7 for illustration). In this study, we quantify health losses using Disability-Adjusted Life Year (DALY) approach recommended by the World Health Organization (WHO)^40,68^. Specifically, the years of life lost due to premature mortality (YLL) are combined with the years of life lived with disability (YLD), producing a single quantity expressing the burden of disease in time units:

3 $$\begin{aligned} \text {DALY} = \text {YLL} + \text {YLD} \end{aligned}$$

For each infected individual (represented by an agent in the ABM), YLL is calculated as the difference between the life expectancy and the year of death if this agent dies as a result of the COVID-19. The second term, YLD, is measured by the duration of the disease within an infected agent who recovers from the COVID-19 (adjusted by a disability weight representing the disease severity). For non-infected agents, $\text {YLL} = 0$ and $\text {YLD} = 0$, under the assumption that the COVID-19 has not affected their health conditions. In this study, we also assumed that a life year lost due to the COVID-19-related death and an impacted year lived with disease for non-fatal cases are equally important (that is, we set the disability weight equal to 1). In addition, no age weighting and discounting on future health benefits^68^ were applied in our calculation for DALY, following^69^ and^70^. The health impacts were calculated at the population level, accumulating the single measurements from all agents in our ABM.

Furthermore, the cost of the evaluated intervention is estimated under the assumption of the equal distribution of the total cost across the agents. When an SD intervention is imposed over a population fraction defined by some SD compliance level, the corresponding cost is assumed to be proportional to this fraction. For example, an intervention with the SD level of 50% is assumed to cost half as much as the full lockdown at the SD level of 100%. A scaling in proportion to the number of impacted individuals is applied in approximating the weekly intervention costs for a typical town, given the intervention costs estimated at $1.4 billion per week for the entire Australian economy (i.e., the entire population)^71^.

The NHB approach allows us to comparatively evaluate the cost-effectiveness of various interventions which may significantly differ in their costs and corresponding health effects. Consequently, it enables to derive adaptive SD interventions by gradually changing the SD levels in a direction that increases the cost-effectiveness. Thus, the NHB can be easily used by a reinforcement learning process exploring the search-space for more cost-effective interventions.

### Willingness to pay

Prior studies considered a broad range of the WTP levels. For example, the cost per quality-adjusted life year (QALY) can be estimated as the probability that the respondent will reject the bid values^72,73^. The estimates by this study resulted in: JPY 5.0 million in Japan (US$41,000 per QALY), KNW 608 million in the Republic of Korea (US$74,000 per QALY), NT 2.1 million in Taiwan (US$77,000 per QALY), $$\pounds$$23,000 in the UK (US$36,000 per QALY), AU$64,000 in Australia (US$47,000 per QALY), and US$62,000 per QALY in the USA.

Another approach determined that, on average, the cost per DALY averted was related to the Gross Domestic Product (GDP) per capita. For instance, the cost was 0.34 times the GDP per capita in low Human Development Index (HDI) countries, 0.67 times the GDP per capita in medium HDI countries, 1.22 times the GDP per capita in high HDI countries, and 1.46 times the GDP per capita in very high HDI countries^74^. For Australia, this would correspond to the cost in the range of AU$93,197.9 = US$67,735.6 (or, 1.22 x US$55521.0) and AU$111,531.9 = US$81,060.7 (or, 1.46 x US$55521.0). These estimates are produced using data from World Bank^75^ and International Monetary Fund^76^ for the average 5-year GDP per capita and USD-AUD exchange rate, respectively.

Another accepted approach is to represent the WTP threshold as the (consumption) value that a society attaches to a QALY^77^. This societal perspective was followed by the contingent valuation approach which valued QALYs under uncertainty for the Dutch population, producing the range from €52,000 to €83,000 (approximately, AU$82,409.9 - AU$131,538.8).

A recent study in the Australian context used a range of WTP up to US$300,000 (or AU$412,771.9) per health-adjusted life year (HALY). It specified preferable COVID-19 intervention policies in three ranges: (i) up to US$20,000 (AU$27,518.1), (ii) from US$30,000 (AU$41,277.2) to US$240,000 (AU$330,217.4), and (iii) above US$240,000^50^.

These studies informed the choice of the WTP thresholds used in our analysis. In particular, we considered three WTP thresholds: $10K per DALY, $50K per DALY and $100K per DALY.

### Agent-based model for COVID-19 transmission and control

In order to simulate transmission and control of the COVID-19 pandemic in Australia we used a well-established ABM^19,31,78^, calibrated to the Delta variant (B.1.617.2), and modified to capture a fluctuating adherence to social distancing as well as more refined vaccination coverage. The original ABM included a large number of individual agents representing the entire population of Australia in terms of demographic attributes, such as age, residence and place of work or education. In re-calibrating and validating this model, we used a surrogate population of New South Wales (7,485,860 agents), while the primary simulations, coupled with the RL algorithm, employed a surrogate population of 2393 agents representing the population of a small Australian local area (e.g., a town), generated to match key characteristics of the Australian census carried out in 2016. The ABM is described in detail in Supplementary Material: Agent-based Model, and here we only summarise its main features.

Each agent belongs to a number of mixing contexts (household, community, workplace, school, etc.) and follows commuting patterns between the areas of residence and work/education. The commuting patterns are obtained from the Australian census and other datasets available from the Australian Bureau of Statistics (ABS)^79–81^. The transmission of the disease is simulated in discrete time steps, with two steps per day: daytime for work/education interactions, and nighttime for all residential and community interactions. The contact and transmission probabilities vary across contexts and ages.

The disease progression within an agent is simulated over several disease-related states, including Susceptible, Infectious (Asymptomatic or Symptomatic), and Removed. All agents are initialised as Susceptible. When an agent is Infectious, other susceptible agents sharing some mixing context with the agent may become infected, and infectious after some latent period. An age-dependent fraction of agents progresses through the disease asymptomatically. The transmission probabilities are determined at each step, given the agents’ mixing contexts, as well as their symptomaticity. The probability of transmission from an Infectious agent varies during the time since the exposure, growing to a peak of infectivity and then declining during the agent’s recovery. At the end of the infectious period, the agents change their state to Removed (i.e., recovered or diseased), which excludes the agent from the Susceptible population. Thus, re-infections are not simulated, given that the simulated timeframe is relatively short (19 weeks following the first week during which the social distancing intervention is triggered, as mentioned below).

A pandemic scenario is simulated by infecting some agents. During calibration and validation, these agents are selected (“seeded”) in proximity to an international airport^19,31^. During the primary simulations of each outbreak in a small Australian town, we seeded all initial cases within this area, according to a binomial sampling process, described in Supplementary Material: Simulation Scenarios and Seeding Method. The seeding process is terminated when cumulative cases exceed a predefined threshold, simulating an imposition of travel restrictions around the town. At this point, the infections may continue to spread only as a result of the local transmission.

A vaccination rollout scheme is implemented in two modes: (i) a progressive rollout mode (i.e., reactive vaccination) used to validate the model with the actual data from the Sydney outbreak during June-November 2021, and (ii) a pre-emptive mode used to simulate pandemic scenarios controlled by NPIs, assuming that some population immunity has been already developed in response to past vaccination campaigns. Both modes assume hybrid vaccinations with two vaccines: Oxford/AstraZeneca (ChAdOx1 nCoV-19) and Pfizer/BioNTech (BNT162b2), concurring with the Australian campaigns during 2021^25,31^.

Different NPIs are simulated: case isolation, home quarantine, and social distancing interventions^19,31^. Case isolation and home quarantine are assumed to be the baseline interventions, activated from the simulation onset. Social distancing (i.e., “stay-at-home” restrictions) is only triggered when cumulative cases surpass a specific threshold. Unlike previous implementations of the ABM, the compliance of agents, bounded by a given SD level, is simulated heterogeneously and dynamically, with Bernoulli sampling used to determine whether an agent is compliant with the SD intervention at any given simulation step (within the total limit on the fraction of compliant agents). Vaccination states and compliance with NPIs modify the transmission probabilities in the corresponding mixing contexts, thus affecting spread of the outbreak.

Importantly, the health effects resulting from the COVID-19 pandemic are captured by aggregating the high-resolution data simulated at the agent level. Unlike other studies which estimate the outcomes only at the end, we quantify the health losses after every simulation day, by measuring probable age-dependent deaths and total probable impacted days for newly infected agents. This temporal resolution allows us to construct a decision process evaluating social distancing interventions in a way compatible with the RL method. Specifically, each decision point includes a state (i.e., information describing the current pandemic situation across all agents), an action (e.g., a decision setting a level of compliance with social distancing below the limit $SD_{max}$), and the associated outcome.

### Reinforcement learning-based search for cost-effective NPIs

**Figure 6:**
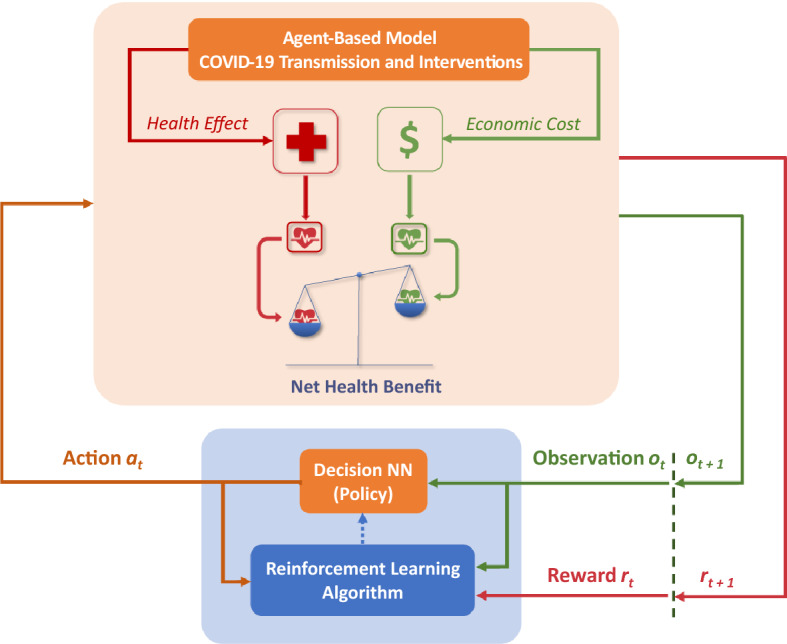
RL-based framework for optimising cost-effectiveness of SD interventions.

Our framework for optimising the cost-effectiveness of SD interventions includes two typical RL components: a decision-maker and an environment, as shown in Fig. 6. The decision-maker is configured as a neural network^82–85^ that can make decisions on the SD compliance levels (within the limit $SD_{max}$), given the decision-maker’s observation of the environment. The environment comprises the ABM which simulates effects of these decisions on the transmission and control of the COVID-19 within a typical Australian town, as described in previous section. Our objective is to learn the decision-making neural network based on the interactions between the decision-maker and the environment, so that cost-effectiveness of the SD intervention is maximised.

In our setting, once the outbreak starts, the decisions are assumed to be made every week, concurring with the time resolution adopted in other studies^55,56,86^. At a decision point *t*, the decision-maker takes a (partial) observation of the environment (denoted by $o_t$), and selects its action $a_t$ aiming to cost-effectively control the ongoing outbreak. An observation characterises the current pandemic state, including the detected incidence (asymptomatic and symptomatic), prevalence, and the count of recoveries and fatalities. Once decision $a_t$ is made setting the SD compliance for the next week, the ABM environment simulates the SD intervention associated with $a_t$ and its effects during the period from the decision point *t* to the next decision point $t+1$. This simulation determines the economic costs incurred during the period (i.e., one week) and the associated health losses (averted DALY). These quantities constitute the reward signal, providing feedback to the decision-maker. At the next decision point this feedback is used to evaluate the choice of $a_t$.

The interactions between the decision-maker and the environment start when the number of cumulative cases exceeds a threshold triggering the SD interventions, and continues until the end of the simulation period (e.g., includes $N = 19$ decision points $t \in \{0, 1, 2,\ldots , N\}$). The interactions form a sequence of observations, actions, and rewards, registered at multiple decision points. The RL algorithm samples from this sequence, perform its optimisation, and updates the decision neural network. In general, this sampling step can be carried out at every decision point as the new data are collected, or be delayed depending on the algorithm.

All interactions between the decision-maker and the environment form an “episode” in the learning process of optimal SD interventions. The learning process involves multiple episodes independently following each other. The total reward generated during an episode, or the episodic reward, is expected to grow as the learning process continues, evidencing that the learned NPIs generate higher NHB. The learning process continues until only minimal improvements in the episodic reward are observed, marking convergence of the RL algorithm. The resultant decision neural network can then be used unchanged during the evaluation phase. The results and analysis presented in section "Results" are based on the evaluation phase.

The process of decision-making follows a Markov Decision Process (MDP) represented by a tuple $\langle S, O , P, A, R \rangle$. The set *S* contains possible states of the environment. The decision-maker is assumed to observe these states only partially, and the set *O* contains all partial observations. The set *A* contains all possible actions $a_t$ available to the decision-maker at each time step *t*. Each action $a_t \in A$ determines the corresponding SD compliance level $f(a_t)$ for the SD intervention, imposed over the population between the time step *t* and the next time step $t+1$. Formally, $f:A \rightarrow [0,1]$, e.g., $f(a^0_t) = 0$ for zero SD intervention $a^0$ at any time *t*. Note that $a_t$ defines the SD intervention applied in addition to baseline interventions, such as CI, HQ and TR which are always enabled by default.

In general, the decision-maker can determine its action according to a stochastic policy $\pi :O \times A \rightarrow [0,1]$ , or a deterministic policy $\pi : O \rightarrow A$. In this study, we configure the decision-maker to follow a stochastic policy described by probability distribution $\pi (o,a)$. Given observation $o_t$ obtained at time step *t*, the action $a_t$ can be sampled from the policy distribution, denoted as $a_t \sim \pi (o_t,\cdot )$.

Unlike other studies^52,56^, which discretise the range of social distancing percentages, we defined $f(a_t)$ to be continuous in the interval $[0, SD_{max}]$, for some limit $0 \le SD_{max} \le 1$. The execution of an action $a_t$ at the state $s_t \in S$ constrains the environment dynamics developing between the time steps *t* and $t+1$. The state transition probability, denoted by $P(s' | s,a): S \times A \times S \rightarrow [0,1]$, quantifies the chance of transition from the current state *s* to the next state $s'$, following the execution of the action *a*. Thus, the probability $P(s' | s,a)$ reflects the pandemic dynamics controlled by the interventions. After the action $a_t$ is executed, the environment produces the reward signal $r_{t+1} \in R$ so that the agent can reinforce its policy at the next time step $t+1$. Each reward $r_{t+1}$ is given by the corresponding health effects attained during the simulated period.

**Figure 7:**
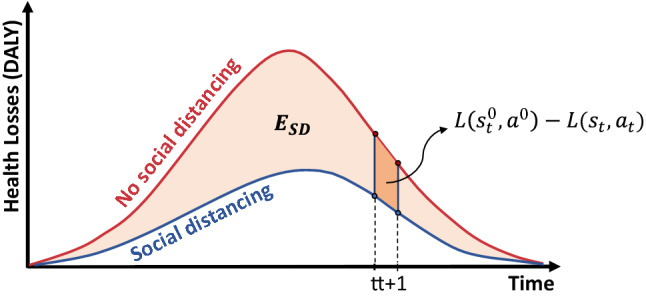
The health effect $E_{SD}$ is shown as the difference between health losses resulting from the null intervention without social distancing (red curve) and health losses averted by a social distancing intervention (blue curve). Period-wise health effect: $E_{SD} = \sum _{t=0}^N \left[ L(s^0_t,a^0) - L(s_t,a_t) \right]$.

In order to optimise the SD interventions by maximising their NHB estimates over the entire simulation period of *N* weeks, we use a period-wise approach maximising the following objective function (see Supplementary Material: Period-wide NHB Objective Function for further details):

4 $$\begin{aligned} \max _{\pi _{\theta }} \ \ \mathop {{\mathbb {E}}}_{\begin{array}{c} a_t \sim \pi _{\theta }(o_t, \cdot ) \\ (s_t, a_t, s_{t+1})\sim\tau \\ (s^0_t, a^0, s^0_{t+1})\sim\tau ^0 \end{array}} \ \ \sum _{t=0}^{N} \left[ L(s^0_t, a^0) - L(s_t, a_t) - \frac{f(a_t) \ C^1}{\lambda } \right] \ , \end{aligned}$$

where $\pi _{\theta }$ is the policy shaped by parameters $\theta$; *L*(*s*, *a*) is the health losses, measured in DALYs, resulting when the intervention at the SD level *a* is applied to the environment at state *s*; action $a_t$ is sampled from policy $\pi _{\theta }(o_t, \cdot )$ based on the environmental observation $o_t$; the transition from $s_t$ to $s_{t+1}$ belongs to the trajectory $\tau$ controlled by SD interventions $a_t$ (i.e., is sampled from a distribution of trajectories); the transition from $s^0_t$ to $s^0_{t+1}$ belongs to the uncontrolled trajectory $\tau ^0$ shaped by null action $a^0$; and $C^1$ is the mean cost for the full 100% SD intervention between two consecutive time steps, with the full cost scaled down by the factor $f(a_t) \in [0, SD_{max}]$. The difference in the health losses between the trajectories $\tau ^0$ and $\tau$, representing the health effects of the simulated SD intervention, is illustrated in Fig. 7.

In order to maximise the objective function expressed by Eq. 4, we specify the reward signal for the action $a_t$ as follows:

5 $$\begin{aligned} r(s_t, a_t | s^0_t) = L(s^0_t, a^0) - L(s_t, a_t) - \frac{f(a_t) \ C^1}{\lambda } \end{aligned}$$

Maximising the total received rewards along the trajectory $\tau$ is equivalent to maximising the objective expressed in Eq. 4, yielding the optimal decision-making policy $\pi ^*$.

The ABM simulation is inherently stochastic, and hence, we use a discounted version of the accumulated rewards:

6 $$\begin{aligned} \max _{\pi _{\theta }} \ \ \mathop {{\mathbb {E}}}_{\begin{array}{c} a_t \sim \pi _{\theta }(o_t, \cdot ) \\ (s_t, a_t, s_{t+1})\sim\tau \\ (s^0_t, a^0, s^0_{t+1})\sim\tau ^0 \end{array}} \ \ \sum _{t=0}^{N} \gamma ^{t} r(s_t, a_t | s^0_t) \end{aligned}$$

where $\gamma \in (0,1)$ is the discount factor, and *r* is the reward function defined by Eq. 5.

The policy $\pi _{\theta }$ is determined by a set of parameters $\theta$ which specify the weights of the decision neural network. A parameterised policy $\pi _{\theta }$ can be optimised by maximising a policy performance measure function $J(\theta )$. A canonical update for the parameter $\theta$ in each learning step *k* follows the gradient ascent method^82^, seeking to maximise the performance function $J(\theta )$:

7 $$\begin{aligned} \theta _{k+1} = \theta _{k} + \alpha \widehat{\nabla J(\theta _k)} \end{aligned}$$

where $\alpha$ is the learning rate for the update, and $\widehat{\nabla J(\theta _k)}$ is the estimation for the gradient of the performance function with respect to $\theta _k$.

In our study, we used the Proximal Policy Optimisation (PPO) algorithm^87^ (see Supplementary Material: PPO Algorithm), aiming to avoid “destructive large policy updates” reported when the discounted objective function, defined by Eq. 6, is optimised directly^87^. Specifically, we utilised the implementation of PPO for continuous actions provided by the Stable-Baselines3 library^88^. The convergence in the training for SD intervention policies, evidenced by improvement of the accumulated rewards over training episodes, is presented in Supplementary Material: Empirical Convergence in the Training of SD Policies.

## Supplementary Information

Supplementary Information.

## Data Availability

Anonymised data used to build the ABM were obtained from the 2016 Australian Census, provided by the Australian Bureau of Statistics (ABS). Vaccination data, including the number of first and second doses administered daily in New South Wales, Australia, between 2 July 2021 and 27 October 2021, were extracted from the COVID-19 Vaccine Roll-out Reports published daily by the Department of Health, Australian Government, during this period. The sources of actual pandemic data for New South Wales: https://data.nsw.gov.au and https://www.covid19data.com.au. Data used and generated by our simulation and optimisation are available on request at Zenodo^89^.

## Code availability

The software code is divided into two main parts: (i) the code validating the ABM against actual pandemic data in New South Wales, and (ii) the code optimising adaptive NPIs using the RL algorithm, for a typical town in New South Wales. Both parts are available on request at Zenodo^89^.
