# Supplementary material for: A general framework for optimising cost-effectiveness of pandemic response under partial intervention measures
Source: Sci Rep. 2022 Nov 14;12:19482. doi: 10.1038/s41598-022-23668-x (PMC9662136; doi:10.1038/s41598-022-23668-x)
Supplement: Supplementary file 1 — Supplementary Information. [file 41598_2022_23668_MOESM1_ESM.pdf]

# A general framework for optimising cost-effectiveness of pandemic response under partial intervention measures

# Supplementary Material

## 1 Agent-based Model

We follow the agent-based modeling approach to simulate the transmission and control of COVID-19 on two scales: (i) a population of 7,485,860 agents to simulate the population of New South Wales, Australia, and (ii) a population of 2,393 agents to simulate the population of a local government area (LGA). The ABM simulates the interactions of agents within different social mixing contexts, with distinct contextual transmission probabilities calibrated to the Delta variant (B.1.617.2) of SARS-CoV-2. The surrogate population is generated based on the Australian Census and related data from the Australian Bureau of Statistics (ABS). The dynamics of the COVID-19 transmission incorporates both non-pharmaceutical interventions, e.g. social distancing, and vaccination rollout schemes. The original ABM, implemented in C++ programming language[1] was extended with new features, including social distancing levels varying over time, two-dose vaccination, and estimation of the net health benefit in terms of the incurred economic costs and associated health effects, and implemented in Python programming language.

### 1.1 Surrogate Population: Demographics

Each agent in the surrogate population is individually simulated to represent an anonymous person with typical attributes, e.g., age, life expectancy, residence, workplace or school, according to the Australian Census, and other ABS datasets, as well as the Australian Curriculum and Assessment and Reporting Authority (ACARA) data. The age of an agent is sampled according to the 2016 ABS data on the estimated resident population by single year of age in New South Wales (NSW), Australia. The agents' life expectancy is assigned according to the 2016-2018 ABS data on life expectancy at birth by state and territory of usual residence. Agents' residences are artificially created based on the census of Statistical Local Areas (SLAs) and Collection Districts (CDs) defined by Australian Standard Geographical Classification (ASGC). Agents are assumed to follow recurrent travel patterns to usual destinations, e.g., workplaces or schools, and are expected to interact within communities and neighborhoods, as well as in their households. During each day, the agents interactions are split between two 12-hour routines: (i) contacts in the workplaces or the schools during daytime, and (ii) contacts in the communities, the neighborhoods, and the households during the nighttime.

Both NSW and LGA populations are generated under the assumption of border closures with nearby states and LGAs respectively. We run the simulation with these surrogate populations to (a) validate the agent-based model with actual data in NSW, and (b) optimise social distancing interventions within a typical LGA, using a deep reinforcement-learning algorithm.

### 1.2 COVID-19 Transmission Model

#### *1.2.1 Transmission Probability*

Transmission of SARS-COV-2 is assumed to be driven by interactions of the agents within their usual working, studying or living contexts. The model runs in discrete time steps (each is a 12-hour cycle) to represent the interactions (i) during the daytime cycle: workplaces (working groups) and schools (classes, grades, schools), and (ii) during the nighttime cycle: neighborhoods, communities, household clusters and households. When a susceptible agent is exposed, within a specific context, to potential infection spread by infectious agent(s), the transmission probability is determined by the infection probability of this context and the age of both susceptible and infectious agents, as described below, following prior studies[1,2].

An agent can be in one of four states: Susceptible, Latent, Infectious (asymptomatic or symptomatic), and Removed (recovered or dead). The set $G_i$ contains all contexts to which agent $i$ belongs. Given a specific mixing context $g \in G_i$, the

instantaneous probability $p^g_{j\to i}(n)$ is the probability that an infectious agent $j$ sharing the context $g$ with susceptible agent $i$ transmits the infection to agent $i$. At the time step $n$, the infection probability for susceptible agent $i$ across the entire context $g$ is then calculated as follows:

$$p^g_i(n) = 1 - \prod_{j \in A_g \setminus \{i\}} (1 - p^g_{j\to i}(n)) \quad (1)$$

where $A_g \setminus \{i\}$ is the list of agents in the context $g$ excluding agent $i$. The instantaneous transmission probability is defined as follows:

$$p^g_{j\to i}(n) = \kappa\, f(n - n_j|j)\, q^g_{j\to i} \quad (2)$$

where $\kappa$ is a global transmission scalar used to calibrate the reproductive number $R_0$, $n_j$ is the time step that agent $j$ becomes infected, and $f(n-n_j|j)$ is a function to characterise the infectivity of agent $j$ over time. For an uninfected agent $j$, $n - n_j < 0$ and $f(n-n_j|j) = 0$. For an infected agent $j$, $n - n_j \geq 0$ and $f(n-n_j|j) \geq 0$. The natural history of the disease $f(n-n_j|j)$ is defined to follow a profile calibrated for B.1.617.2 (Fig. 1), as described in prior work[2]. Asymptomatic agents are modeled to be less infectious than symptomatic agents by a factor denoted by $\alpha_{\text{asymp}}$. Lastly, $q^g_{j\to i}$ is the daily probability of transmission from agent $j$ to agent $i$ at the agent $j$'s peak infectivity. The values of $q^g_{j\to i}$ in different mixing contexts are set in accordance to prior studies[1,2] and specified in Table 1.

| Contact Group | Type of Contact | Daily Transmission Probability ($q^g_{j\to i}$) |
|---|---|---|
| Household (size 2) | Any to child (0 - 18) | 0.09335 |
| | Any to adult (19+) | 0.02420 |
| Household (size 3) | Any to child (0 - 18) | 0.05847 |
| | Any to adult (19+) | 0.01495 |
| Household (size 4) | Any to child (0 - 18) | 0.04176 |
| | Any to adult (19+) | 0.01061 |
| Household (size 5) | Any to child (0 - 18) | 0.03211 |
| | Any to adult (19+) | 0.00813 |
| Household (size 6) | Any to child (0 - 18) | 0.02588 |
| | Any to adult (19+) | 0.00653 |
| Household Cluster | Child (0 - 18) to child (0 - 18) | 0.00400 |
| | Child (0 - 18) to adult (19+) | 0.00400 |
| | Adult (19+) to child (0 - 18) | 0.00400 |
| | Adult (19+) to adult (19+) | 0.00400 |
| Working Group | Adult (19+) to adult (19+) | 0.00400 |
| School | Child (0 - 18) to child (0 - 18) | 0.00029 |
| Grade | Child (0 - 18) to child (0 - 18) | 0.00158 |
| Class | Child (0 - 18) to child (0 - 18) | 0.00865 |
| Neighborhood | Any to child (0 - 4) | $0.035 \times 10^{-5}$ |
| | Any to child (5 - 18) | $1.044 \times 10^{-5}$ |
| | Any to adult (19 - 64) | $2.784 \times 10^{-5}$ |
| | Any to adult (65+) | $5.568 \times 10^{-5}$ |
| Community | Any to child (0 - 4) | $0.872 \times 10^{-6}$ |
| | Any to child (5 - 18) | $2.608 \times 10^{-6}$ |
| | Any to adult (19 - 64) | $6.960 \times 10^{-6}$ |
| | Any to adult (65+) | $13.92 \times 10^{-6}$ |

**Table 1.** Daily transmission probability $q^g_{j\to i}$ from infected agent $j$ to susceptible agent $i$ in different contact groups. Numbers in brackets define age groups to categorise children or adults.

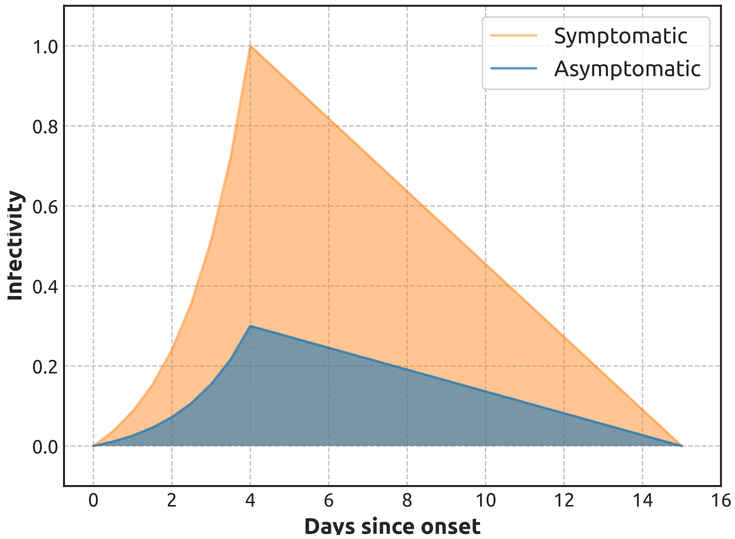


**Figure 1.** Simulated natural history of disease (calibrated to B.1.617.2). The agent infectivity is assumed to be exponentially increasing until its peak ($f(n-n_j|j)=1.0$). In the post-peak period, the infectivity level linearly decreases to 0.0, the moment marking transition to Removed state.

Finally, we derive the infection probability for agent $i$ at the time step $n$ across all shared contexts $G_i$:

$$\begin{aligned} p_i(n) &= 1 - \prod_{g \in G_i(n)} \left(1 - p_i^g(n)\right) \\ &= 1 - \prod_{g \in G_i(n)} \prod_{j \in A_g \setminus \{i\}} \left(1 - p_{j \to i}^g(n)\right) \end{aligned} \tag{3}$$

Bernoulli sampling, based on $p_i(n)$, is then used to determine whether a susceptible agent $i$ becomes infected at the end of each time step.

Since an infection can eventually lead to a symptomatic or asymptomatic case, we adjust the infection probability quantified by Eq. 3 by an additional factor $\sigma$ representing the fraction of symptomatic cases over the total cases:

$$p_i^d(n) = \sigma(i)\ p_i(n) \tag{4}$$

where $\sigma(i)$ is a piecewise function dependent on the age of agent $i$. Specifically, $\sigma(i)$ is defined by a simple assignment of two values specified for adults (age $\geq 18$), $\sigma_a = 0.67$, and children (age $< 18$), $\sigma_c = 0.268$, see[2].

#### *1.2.2 Recovery and Fatality*

We use a truncated age-specific infection fatality rate (IFR) estimation[3], with a scaling adjustment for B.1.617.2[4], to determine the probability of death for an infected agent:

$$\text{IFR}(age) = \min\left(0.1, 0.0232 \times 10^{-3.27+0.0524 \times age}\right) \tag{5}$$

Given a new infection, Bernoulli sampling is used, according to IFR expressed by Eq. 5, to determine whether the agent will recover or die at the end of the disease progression (in Removed state). The outcome is then used to calculate the health effect for this agent, measured as disability-adjusted life years, i.e. DALYs. The effect of a death is estimated by the difference between the agent's current age and its expected life expectancy. The health loss of a recovered case is the onset-to-recovery time, estimated by a gamma distribution with the mean of 24.7 days and the coefficient of variation of 0.35, following[5].

### 1.3 Non-Pharmaceutical Interventions

We model several non-pharmaceutical interventions (NPIs), e.g., case isolation (CI), home quarantine (HQ), school closure (SC), and social distancing (SD). Each NPI is modelled in terms of its compliance level, defined as the fraction of the population complying with this NPI, as well as the adjusted strength of interactions between a compliant agent and other agents sharing

their mixing groups. The infection probability $p_i(n)$ for compliant agents is adjusted to account for the NPIs effects as follows:

$$p_i(n) = 1 - \prod_{g \in G_i(n)} \prod_{j \in A_g \setminus \{i\}} (1 - F_g(j)\, p^g_{j \to i}(n)) \tag{6}$$

where $F_g(j) \neq 1$ is the strength of the interaction between agent $j$ and other agents in the shared context $g$. For non-compliant agents $j$, the interaction strength is unchanged: $F_g(j) = 1$.

When agent $j$ complies with multiple NPIs, the value of $F_g(j)$ is preferentially assigned to only one NPI in accordance with the following order: CI, HQ, SD, SC. For example, if an infected agent is compliant with both CI and SD, its associated interaction strength is set according to CI: $F_g(j) = F^{\mathrm{CI}}_g(j)$ as case isolation takes precedence over other interventions. Table 2 summarises the values of $F_g(j)$ for different interventions, as well as the baseline compliance levels of these interventions. Given these compliance levels, at each time step, the compliant and non-compliant agents are randomly selected for each NPI according to Bernoulli process. While the compliance levels for CI, HQ, and SC are fixed in the simulation, the compliance level for SD (in short, the SD level) is controlled by a decision-making policy.

| | **Intervention** | | | | |
|---|---|---|---|---|---|
| **Mixing Group** | *CI* | *HQ* | *SD* | *SC (parent)* | *SC (child)* |
| Household | 1.0 | 2.0 | 1.0 | 1.0 | 1.0 |
| Household Cluster | 0.25 | 0.25 | 0.25 | 0.5 | 0.5 |
| Working Group | 0.25 | 0.25 | 0.1 | 0 | - |
| School/Grade/Class | 0.25 | 0.25 | 0.1 | - | 0 |
| Neighborhood (CD) | 0.25 | 0.25 | 0.25 | 0.5 | 0.5 |
| Community (SLA) | 0.25 | 0.25 | 0.25 | 0.5 | 0.5 |
| **Compliance Level** | 0.7 | 0.5 | - | 0.25 | 1.0 |

**Table 2.** Interaction strengths and compliance levels for the considered NPIs across different mixing contexts.

## 1.4 Vaccination Strategy and Vaccine Efficacy

### *1.4.1 Vaccination Strategy*

We simulate two vaccination rollout strategies in order to (i) validate our model against the actual pandemic data in NSW during an outbreak of the Delta variant over June-November 2021, and (ii) optimise adaptive SD interventions against future outbreaks within a local government area. The first objective follows (i) progressive (reactive) vaccination strategy, capturing the vaccination dynamics in NSW in 2021, while the second objective is modelled with (ii) pre-emptive vaccination strategy.

**Progressive vaccination.** Approximating the actual vaccination campaign in NSW, the progressive rollout is modelled as a hybrid approach with two types of vaccines: Oxford/AstraZeneca (ChAdOx1 nCoV-19) and Pfizer/BioNTech (BNT162b2). Our simulation closely matches the number of first and second doses administered daily in NSW. The actual numbers are extracted from multiple COVID-19 vaccine rollout reports published daily by the Department of Health, Australian Government from 01 July 2021 to 27 October 2021[6], as shown in Fig. 2. We also assume an equal distribution of ChAdOx1 nCoV-19 and BNT162b2 for individuals aged 16 and over, following another vaccine safety report from the Therapeutic Goods Administration (TGA) of the Department of Health, Australian Government, which specified that 12 million BNT162b2 doses and 10.8 million ChAdOx1 nCoV-19 were administered by 12 September 2021[7]. For individuals aged 12-15, BNT162b2 is assumed to be the only administered vaccine, again in concordance with the adopted practice. In addition, for each agent, our simulated rollout strategy uses the same vaccine for dose 1 and dose 2, also in agreement with the practice in 2021. Our age-stratified daily vaccine allocation strategy for different age-groups (12-15, 16-49, 50-69, and 70+) is designed to satisfy these constraints.

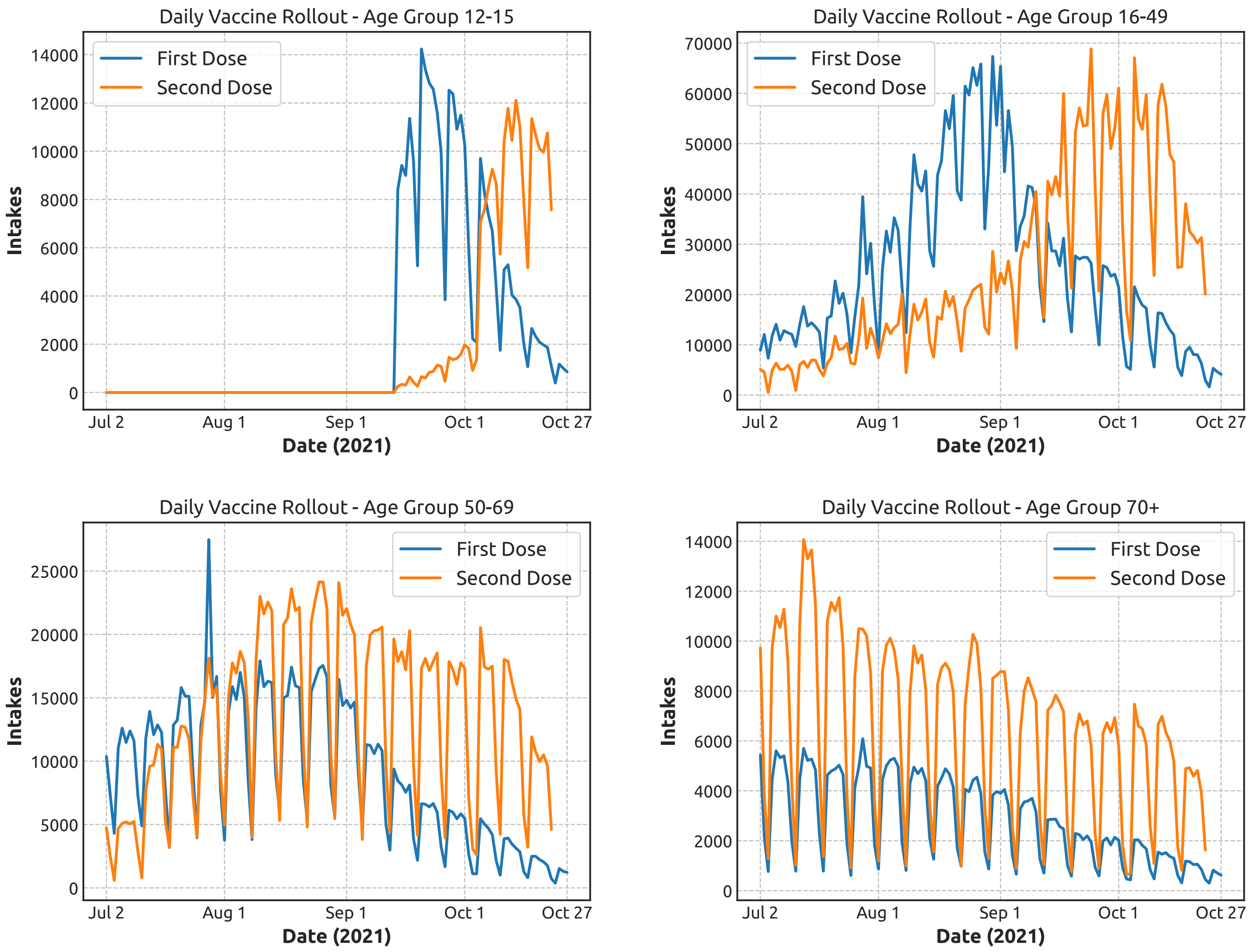


**Figure 2.** Number of first and second doses administered daily in New South Wales, Australia between 2 July 2021 and 27 October 2021. Source data are extracted from COVID-19 Vaccine Roll-out Reports published daily by the Department of Health, Australian Government during this period[6].

**Pre-emptive vaccination.** Under this strategy, vaccine is rolled out before a pandemic, aiming to provide a significant portion of the population with some immunity. In our study, before the first simulation time step, two-dose vaccination is assigned to 85% of adults and children. Pre-emptive vaccination also assumes to follow a hybrid approach combining two vaccines: ChAdOx1 nCoV-19 and BNT162b2, with an approximately equal number of these two vaccines for individuals aged 16 and over, and predominantly BNT162b2 vaccine for individuals under 15 years of age.

### *1.4.2 Vaccine Efficacy*

Following[2,8], our model is designed with different components of vaccine efficacy: efficacy against susceptibility (VEs), efficacy against susceptibility to disease (VEd), efficacy against infectiousness (VEi), as well as efficacy against death (VEp). Unlike[2,8], during the progressive vaccination, we vary these efficacy components over time, following a profile shown in Fig. 3. Vaccine efficacy is assumed to increase linearly from the days when the agent takes the doses (first, D1, or second, D2) and reach the maximum level of protection after a certain delay (Max_D1 and Max_D2 for first and second dose respectively). After taking the first dose, agents also need to wait a certain period of time (Min_Delay) before registering for the next shot. Summary of our settings for these parameters is given in Table 3 for ChAdOx1 nCoV-19 and BNT162b2. For the pre-emptive vaccination strategy, we assume that all vaccinated agents get their both doses before an outbreak, and have sufficient time to build their full immunity against the COVID-19. The vaccine efficacy is assumed to be sustained at its highest level, once it is attained (which is realistic to assume for relatively short simulation horizons of approximately 20 weeks).

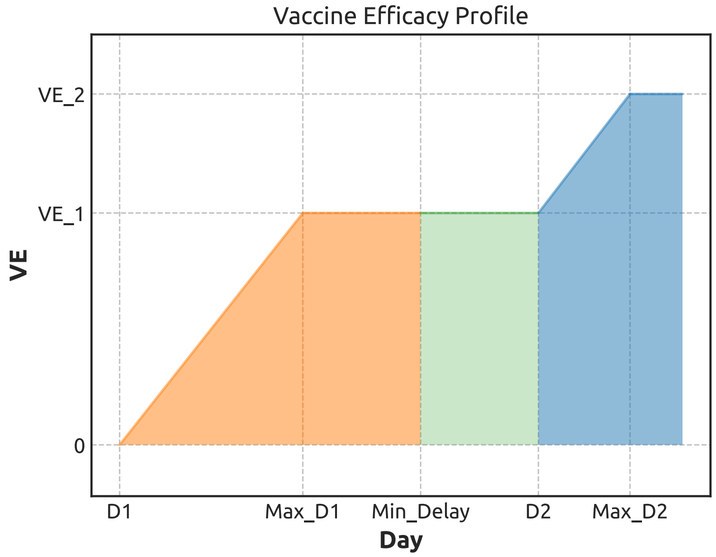


**Figure 3.** Vaccine Vaccine efficacy profile assumed for a two-dose vaccine rollout during the period between June 2021 and October 2021 in NSW, Australia. VE_1 and VE_2 denote the maximum vaccine efficacy after dose 1 and dose 2, respectively. D1 and D2 denote the day of the first and second dose, while Max_D1 and Max_D2 denote the day when the maximum vaccine efficacy is reached for each dose. Min_Delay denotes the delay between the doses.

| VE Parameter | Description | ChAdOx1 nCoV-19 | BNT162b2 |
|---|---|---|---|
| D1 | Time when the first dose is taken | - | - |
| Max_D1 | Time to build full immunity after the first dose | D1 + 21 days | D1 + 14 days |
| Min_Delay | Minimum time between the first and second doses | 28 days | 21 days |
| D2 | Time when the second dose is taken | - | - |
| Max_D2 | Time to build full immunity after the second dose | D2 + 14 days | D2 + 7 days |

**Table 3.** Parameters setting the vaccine efficacy profile for ChAdOx1 nCoV-19 and BNT162b2.

Given vaccination components VEs and VEi, the infection probability defined by Eq. 6 is adjusted as follows:

$$p_i(n) = (1 - \mathrm{V}(\mathrm{VEs}_{max}, n, n_i^v)) \left(1 - \prod_{g \in G_i(n)} \prod_{j \in A_g \setminus \{i\}} \left(1 - (1 - \mathrm{V}(\mathrm{VEi}_{max}, n, n_j^v))\ F_g(j)\ p^g_{j \to i}(n)\right)\right) \tag{7}$$

where $n_i^v$ and $n_j^v$ are the time steps when agent $i$ and agent $j$ take their latest vaccine shots, $\mathrm{V}(\cdot)$ is a function returning vaccine efficacy values defined by the profile shown in Fig. 3, $\mathrm{VEs}_{max}$ and $\mathrm{VEi}_{max}$ are the maximum vaccine efficacies of the latest vaccine dose (dose 1 or dose 2) against susceptibility and infectiousness. For unvaccinated agents, Eq. 7 simplifies with $V(\cdot) = 0$.

The vaccine efficacy against disease (or illness), VEd, affects the probability of generating a symptomatic case, denoted $p_i^d(n)$ for agent $i$. Eq. 4 is adjusted as follows:

$$p_i^d(n) = (1 - V(\mathrm{VEd}_{max}, n, n^v))\, \sigma(i)\ p_i(n) \tag{8}$$

where $\sigma(i)$ is a piece-wise function dependent on the age of agent $i$, setting the fraction of symptomatic agents.

Given the vaccination component VEp, the infection fatality rate of an infected agent at the time step $n$ is modified as follows:

$$\mathrm{IFR}(age, n) = (1 - V(\mathrm{VEp}_{max}, n, n^v))\ \min\left(0.1, 0.0232 \times 10^{-3.27 + 0.0524\ age}\right) \tag{9}$$

where $\mathrm{VEp}_{max}$ is the maximum vaccine efficacy against death attained after the 2nd dose.

Following[8], the values of the vaccine efficacy components are assigned to match the clinical efficacy (VEc) against B.1.617.2[9], constrained as follows:

$$\begin{aligned} \text{VEc} &= 1-(1-\text{VEd})(1-\text{VEs}) \\ &= \text{VEs}+\text{VEd}-\text{VEs}\times\text{VEd} \end{aligned} \tag{10}$$

We solve Eq. 10 for their central estimated values: $\text{VEs} = \text{VEd} = 1-\sqrt{1-\text{VEc}}$. For BNT162b2, under assumption that $\text{VEc} = 0.7$ after dose 1, and $\text{VEc} = 0.9$ after dose 2, we set $\text{VEs} = \text{VEd} = 0.452$ after dose 1 and $\text{VEs} = \text{VEd} = 0.684$ after dose 2. For ChAdOx1 nCoV-19, under assumption that $\text{VEc} = 0.6$ after dose 1 and dose 2, we set $\text{VEs} = \text{VEd} = 0.368$.

For the assignment of VEi, we assume the efficacy against onward transmission to be 0.45 after dose 1 and 0.5 after dose 2 for both types of vaccines[10]. The vaccine efficacy against death is set as $\text{VEp} = 0.71$ (after dose 1) and $\text{VEp} = 0.92$ (after dose 2) for BNT162b2, and $\text{VEp} = 0.69$ (after dose 1) and $\text{VEp} = 0.90$ (after dose 2) for ChAdOx1 nCoV-19[11].

### 1.5 Simulation Scenarios and Seeding Method

We simulate two different scenarios: (i) an outbreak in NSW developing alongside progressive vaccination, as well as NPIs including CI, HQ, SC and SD, and (ii) a potential outbreak in an SLA where 85% of the population have been vaccinated (pre-emptive vaccination). In scenario (ii), adaptive SD interventions are optimised by RL algorithm, while CI and HQ are always enabled as baseline measures. In both simulation scenarios, the outbreak starts once the first infections are 'seeded'.

For scenario (i), we seed the initial infections in the proximity of the Sydney International airport. Each simulated day, new infections are generated in the SLAs within a 50-kilometer radius of the Sydney's international airport, following a binomial distribution dependent on the average daily number of incoming passengers[2]. For scenario (ii), we seed initial infection within the considered SLA.

The seeding continues until a threshold for cumulative cases, the travel restrictions (TR) threshold, is exceeded. During this seeding stage, only vaccination and baseline NPIs (CI, HQ) are simulated. The SD interventions start only when another threshold of cumulative cases, the SD intervention threshold, is exceeded. For scenario (i), the TR threshold is set at 20, while the SD intervention threshold is set at 400 cases. For scenario (ii), both TR and SD intervention thresholds are set at 5.

### 1.6 Model Calibration

We calibrated the model to simulate the transmission of B.1.617.2 variant. By varying ABM parameters, we explored a range of the reproduction number ($R_0$), aiming to attain $R_0$ at least twice as high as the reproduction number of the original SARS-CoV-2 variant[12]. For model validation based on NSW data, we aimed for $R_0$ to stay in the approximate range between 5.3 and 6.5. This range concurs with the $R_0$ estimates for B.1.617.2 in the broad range, 3.2–8.0, reported previously[13,14], as well as the narrow range, 6.0–6.20, used in the Australian study with a similar ABM[2]. For optimisation of NPIs in an LGA, we aimed for $R_0$ to stay in a higher range between 7.0 and 8.0, in order to reflect a higher infectivity of variants causing future outbreaks.

$R_0$ is measured by the expected number of direct secondary cases from a typical infection in a entirely susceptible population[15]. In our ABM, in order to derive $R_0$, we randomly select an agent as the primary case, simulate transmissions, and count all direct secondary cases detected by the simulation. This process is repeated several hundred times.

In order to eliminate the bias in selecting the primary case, we followed "the attack rate pattern weighted index case" method[16,17] based on the age-specific attack rates [0.068, 0.173, 0.140, 0.461, 0.157] determined by the overall simulation for specific age groups: [0-4, 5-18, 19-29, 30-64, 65+]. The resultant value of $R_0$ for the validation scenario was 6.348 (95% CI 5.858–6.839, $N = 300$). For the optimisation scenario with higher infectivity, the resultant value of $R_0$ was 7.582 (95% CI 7.457–7.706, $N = 4,416$), as detailed in subsection 1.7.5.

Many studies have suggested that children are affected less severely by SARS-COV-2 than adults[18,19]. In our ABM, the probability of becoming a symptomatic case is age-dependent. The fraction of symptomatic cases in children was calibrated to be 60% less than adults: $\sigma_{child} = 0.268$, while $\sigma_{adult} = 0.67$. The attack rate for children (under 18), simulated under $\sigma_{child} = 0.268$ without SD interventions, reached 0.24. This rate is in the range of [0.22, 0.29] reported by the National Centre for Immunisation Research and Surveilance (NCIRS) for Sydney outbreak in 2021[20].

The set of calibrated parameters is summarised in Table 4.

### 1.7 Sensitivity Analysis

In order to check robustness of the ABM, we performed a local point-based sensitivity analysis. The analysis quantified the response of specific output variables to changes in key input parameters which were varied while keeping other input variables at their default values. As described in Section 1.6, the default values were calibrated using the data of the third COVID-19 wave which started in NSW in June 2021. The following input parameters were investigated: SD intervention threshold, global transmission scalar ($\kappa$), infectious period ($T_{\text{inf}}$), the fraction of symptomatic cases in children ($\sigma_{child}$), and the reduction in infectivity of asymptomatic cases (i.e., asymptomatic infectivity $\alpha_{asymp}$). The peak incidence and the total fatalities were

| Parameters | Description | Value |
|---|---|---|
| $\kappa$ | Global transmission scalar in Eq. 2 | 6.0 |
| $\sigma$ | Probability that an infection becomes a symptomatic case in Eq. 4 and 8 | 0.67 (for adults)<br>0.268 (for children) |
| $\alpha_{\text{asymp}}$ | Factor for the reduction in transmissibity of an asymptomatic case as shown in Fig. 1 | 0.3 (validation)<br>0.5 (optimisation) |
| $T_{\text{lat}}$ | Latent period | 0 |
| $T_{\text{inc}}$ | Incubation period | 4 days |
| $T_{\text{inf}}$ | Infectious period | 15 days |

**Table 4.** Calibrated ABM parameters.

selected as the output variables. Figures 4–8 traced responses of the output variables with respect to changes in the input parameters. This sensitivity analysis used the simulations for scenario (i), as described in Section 1.5. For each value of the input parameter, we independently simulated a fixed SD intervention specified at different levels: 30%, 40%, or 50%, that is, $SD = 0.3$, $SD = 0.4$, or $SD = 0.4$. Other input parameters, unless varied themselves, are set at their default values determined by the calibration.

#### *1.7.1 SD Intervention Threshold*

The threshold of cumulative cases which triggers the SD interventions is an important input parameter shaping pandemic response. The outbreak in NSW started on 16 June 2021, following a long period without any confirmed locally acquired cases (more than a month since 5 May 2021[21]). The stay-at-home orders with various levels of restrictions were progressively issued since late June 2021. Since the outbreak kept escalating, a tighter lockdown was announced in NSW on 9 July 2021[22], when cumulative incidence reached 449 cases (detected between 16 June 2021 and 8 July 2021). Following this real-world account, we varied the SD intervention threshold in the range between 50 and 450 cases, with an increment step of 50. Figure 4 traces the output variables, i.e. peak incidence and total fatalities, over the simulation period of 114 days. For each simulated SD level (0.3, 0.4, 0.5), the peak incidence and the number of total fatalities are observed to grow with the increase in the SD intervention threshold from 50 to 450.

For $SD = 0.3$, the peak incidence increases 4.06 times from the median value 866.5 (first quartile: 626.25, third quartile: 1240) to the median value 3514 (first quartile: 3305.25, third quartile: 3868.5). The total fatalities increase 4.36 times from the median value 496 (first quartile: 366.75, third quartile: 706.75) to the median value 2162.5 (first quartile: 1986, third quartile: 2284).

For $SD = 0.4$, the peak incidence increases 4.19 times from the median value 367.5 (first quartile: 344, third quartile: 550.5) to the median value 1540.5 (first quartile: 1380, third quartile: 1740). The total fatalities increase 4.5 times from the median value 218 (first quartile: 204, third quartile: 343.25) to the median value 981.5 (first quartile: 891.5, third quartile: 1098).

For $SD = 0.5$, the peak incidence increases 5.64 times from the median value 169 (first quartile: 141.75, third quartile: 204.5) to the median value 952.5 (first quartile: 831.25, third quartile: 1009.5). The total fatalities increase 6.83 times from the median value 89 (first quartile: 65.25, third quartile: 125) to the median value 608 (first quartile: 558.5, third quartile: 668.75).

In summary, a 9-fold increase in the SD intervention threshold linearly leads to an approximately 4 to 7 times increase in the output variables (peak incidence and total fatalities) across all simulations with fixed SD levels (30%, 40%, and 50%). While this sensitivity is higher for lower value $SD = 0.3$, it remains moderate for higher considered SD levels, as shown in Fig. 4. Under $SD = 0.4$ or $SD = 0.5$, i.e., the compliance levels which have been retrospectively estimated for NSW at the time[2], the observed sensitivity markedly diminishes for the SD intervention thresholds which exceed 300 cases and approac the threshold used in NSW (450 cases). This shows that the model is broadly robust to changes in the threshold, with the robustness strengthening in the policy-relevant range.

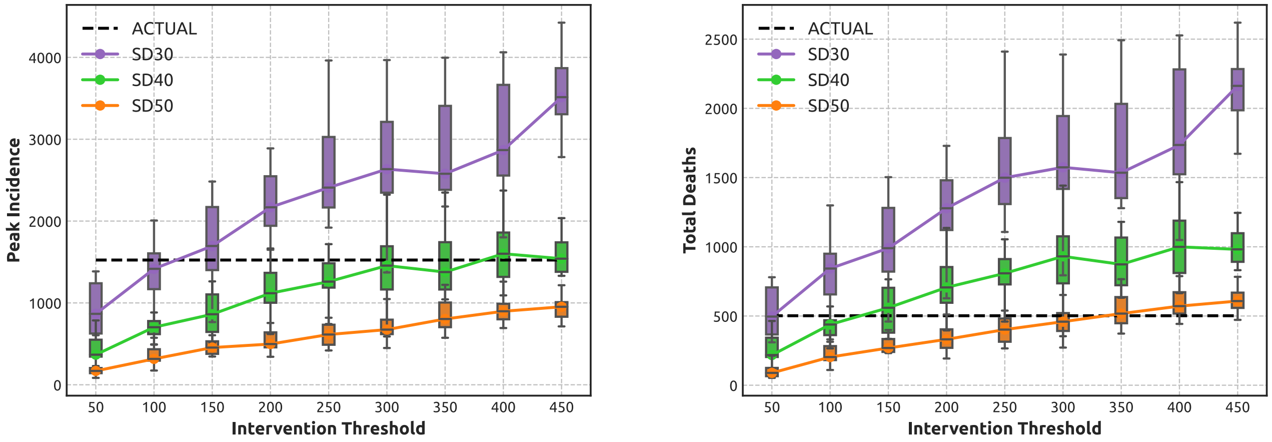


**Figure 4.** Local sensitivity analysis of the output variables (peak incidence and total number of fatalities) with respect to changes in the SD intervention threshold. The black dashed lines trace the actual peak incidence and the total number of fatalities in NSW during the simulated period.

#### 1.7.2 Global Transmission Scalar

The global transmission scalar ($\kappa$, see Eq. 2) was varied around its default value of 6.0, which resulted from the calibration process, as described in section 1.6. We varied $\kappa$ in the range between 5.6 and 6.4, with the increment step of 0.2. The simulated SD interventions (0.3, 0.4, 0.5) are all triggered at the threshold of 400 cumulative cases. Figure 5 shows the corresponding changes in the peak incidence and total fatalities.

For $SD = 0.3$, with the grow of $\kappa$, the peak incidence increases by 253.04% relative to the peak incidence obtained at the lower bound $\kappa = 5.6$ (median 1663, first quartile: 1540.75, third quartile: 1819.5). Similarly, the total fatalities increase by 227.63% relative to the fatalities simulated at the lower bound $\kappa = 5.6$ (median 1046, first quartile: 922.5, third quartile: 1147).

For $SD = 0.4$, the peak incidence linearly increases by 226.69%, and the total fatalities linearly increase by 193.18%.

For $SD = 0.5$, the peak incidence almost linearly increases by 206.57%, and the total fatalities almost linearly increase by 192.71%.

In summary, a 14.3% growth in $\kappa$ from 5.6 to 6.4 increases the outputs approximately 2 to 2.5 times, indicating moderate to high sensitivity, as expected for the global transmission scalar which directly affects the reproduction number $R_0$. Nevertheless, the reported dependencies are linear within the policy-relevant range of $SD = 0.4$ to $SD = 0.5$, and the model is robust in the proximity to the default value $\kappa = 6.0$.

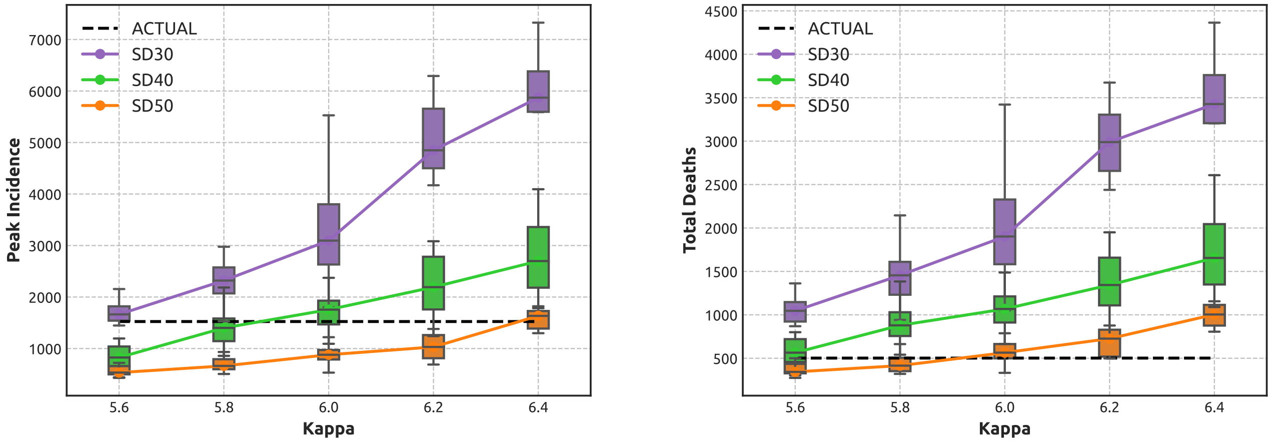


**Figure 5.** Local sensitivity analysis of the output variables (peak incidence and total number of fatalities) with respect to changes in the global transmission scalar ($\kappa$). The black dashed lines trace the actual peak incidence and the total number of fatalities in NSW during the simulated period.

### *1.7.3 Duration of Infectious Period*

The infectious period $T_{\text{inf}}$ spans the entire natural history of the disease, with the infectivity rising to its peak and then linearly reducing to zero, see Fig. 1. We explored the sensitivity of the two output variables with respect to changes in the duration for infectious period which was varied around the calibrated value (15 days). The comparison between the periods of 11, 13, 15 and 17 days was performed for $R_0 \approx 6.35$ (i.e., $\kappa = 6.0$), and the SD intervention threshold set at 400 cases, across three SD compliance levels (0.3, 0.4, 0.5). While varying the infectious period, we fixed the incubation period at 4 days, thus changing only the post-incubation period which was varied as 7, 9, 11 and 13 days.

Figure 6 shows a high sensitivity of the output variables (peak incidence and total fatalities) to the changes in the infectious period. For $SD = 0.3$, the peak incidence produced by $T_{\text{inf}} = 11$ days has the median value 452 (first quartile: 390.25, third quartile: 495.75), and increases dramatically by 1076.88% at $T_{\text{inf}} = 17$ days. The number of total fatalities increases by 984.91% (17 days) relative to fatalities at $T_{\text{inf}} = 11$ days (median: 275, first quartile: 215.25, third quartile: 327.5). Smaller but still significant sensitivities are also observed for $SD = 0.4$ and $SD = 0.5$. These observations limit the model robustness to changes in the duration of infectious period within a narrow range around $T_{\text{inf}} = 15$ and within the policy-relevant range of $SD = 0.4$ to $SD = 0.5$. The observed sensitivity to $T_{\text{inf}}$ highlights the impact of the infectious period's duration on the pandemic scale, especially under modest social distancing levels.

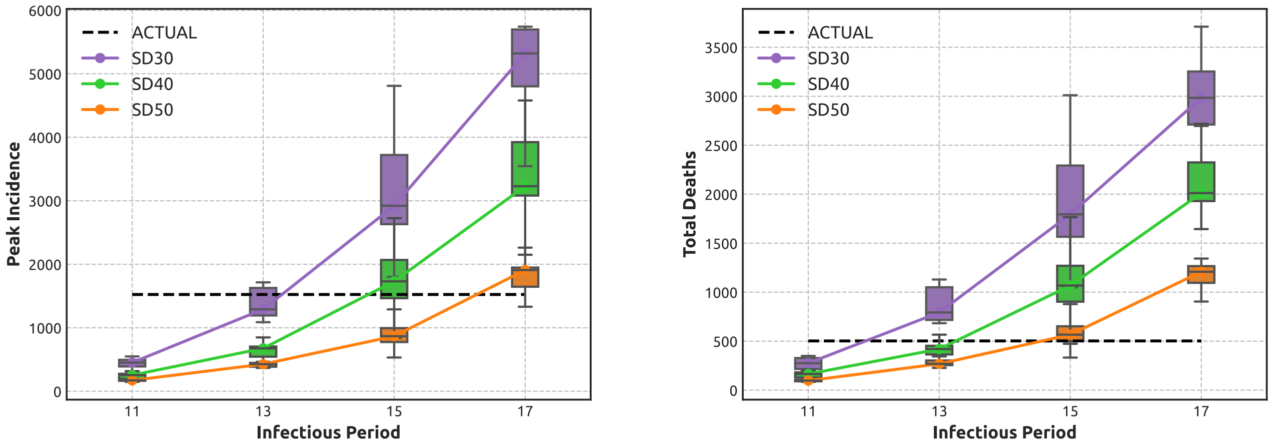


**Figure 6.** Local sensitivity analysis of the output variables (peak incidence and total number of fatalities) with respect to changes in the infectious period ($T_{\text{inf}}$). The black dashed lines trace the actual peak incidence and the total number of fatalities in NSW during the simulated period.

#### *1.7.4 Fraction of Symptomatic Cases in Children*

As the fraction of detected cases in children tends to increases with the B.1.617.2 variant compared to the original variant[20,23], we investigated the sensitivity to $\sigma_{child}$. This input parameter was varied in the range [0.067, 0.268], with the increment step of 0.067.

Figure 7 shows that, as the fraction $\sigma_{child}$ increases, the peak incidence and the number of total fatalities do not exhibit much sensitivity for any SD compliance level, from $SD = 0.3$ to $SD = 0.5$. Across the entire range $\sigma_{child}$, i.e., [0.067, 0.268], the differences between the first and third quartiles of all boxplots (for a specific SD level) are relatively small, with the boxplots nearly overlapping. The low sensitivity of the outputs to changes in $\sigma_{child}$ confirms model robustness with respect to changes in the fraction of detected cases in children.

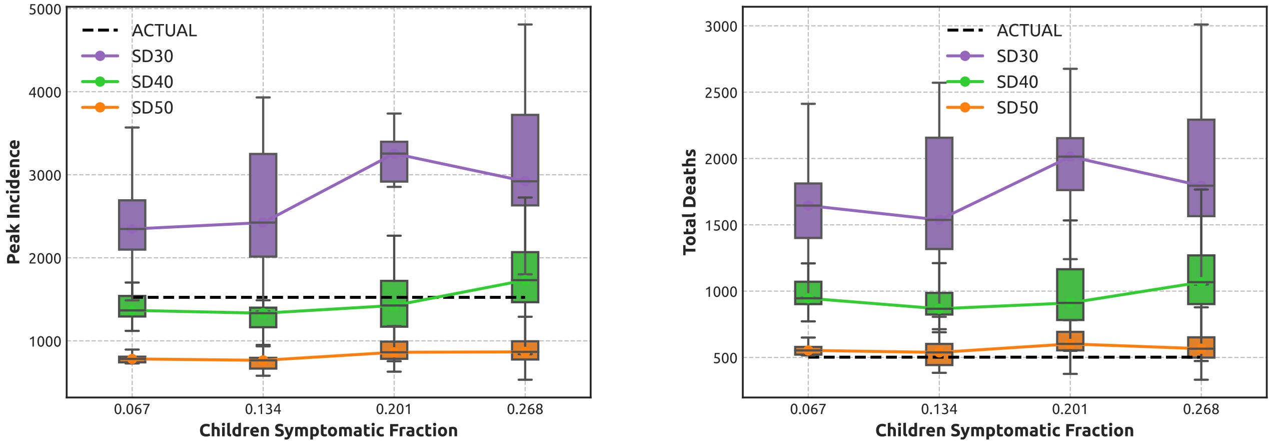


**Figure 7.** Local sensitivity analysis of the output variables (peak incidence and total number of fatalities) with respect to changes in the child symptomatic fraction ($\sigma_{child}$). The black dashed lines trace the actual peak incidence and the total number of fatalities in NSW during the simulated period.

#### *1.7.5 Asymptomatic Infectivity*

In our model, asymptomatic cases are modelled as less infectious than symptomatic cases. The relevant parameter, asymptomatic infectivity $\alpha_{asymp}$, is the fraction specifying the infectivity of a typical infected asymptomatic case in comparison to the maximum level of infectivity in a typical symptomatic case (Fig. 1). We varied $\alpha_{asymp}$ within the range [0.2, 0.5], with increment step

0.1. As shown in Fig. 8, the change in asymptomatic infectivity affects both output variables in all scenarios from $SD = 0.3$ to $SD = 0.5$.

For $SD = 0.3$, the peak incidence at the lower bound $\alpha_{asymp} = 0.2$ has the median value 918.5 (first quartile: 841.5, third quartile: 1023.75), and increases by 1617% when this parameter reaches the upper bound $\alpha_{asymp} = 0.5$. The number of total fatalities starts with the median value 556 (first quartile: 536.75, third quartile: 641) at the lower bound $\alpha_{asymp} = 0.2$, and increases by 1478.33% at the upper bound $\alpha_{asymp} = 0.5$.

For $SD = 0.4$ to $SD = 0.5$, however, the changes in output variables are less significant (about two and three times less, respectively). This points to the model robustness within the policy-relevant range of $SD = 0.4$ to $SD = 0.5$.

Optimisation scenarios used an upper bound $\alpha_{asymp} = 0.5$, to reflect potentially higher asymptomatic infectivity. This setting corresponds to $R_0 = 7.582$, with 95% CI 7.457–7.706, $N = 4,416$.

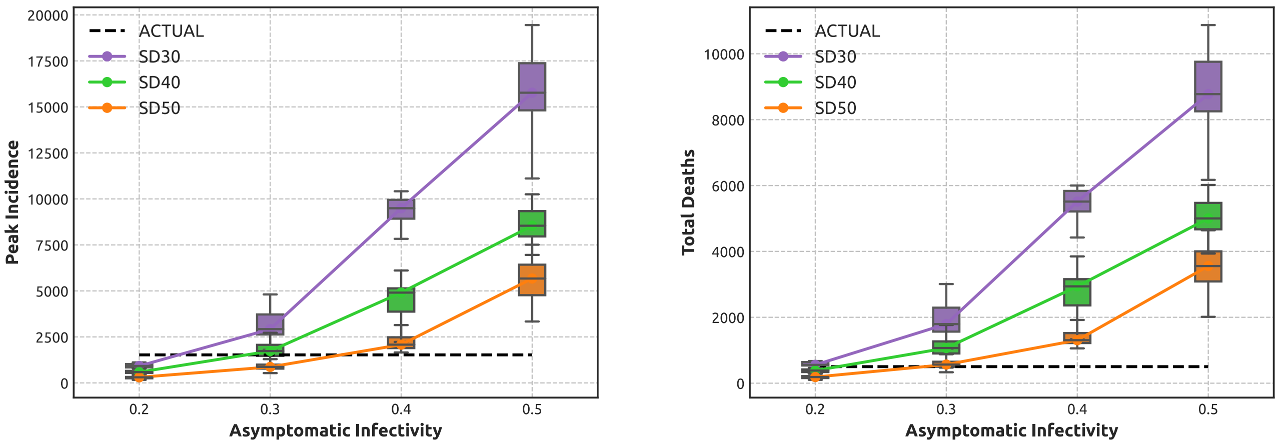


**Figure 8.** Local sensitivity analysis of the output variables (peak incidence and total number of fatalities) with respect to changes in the infectivity of asymptomatic cases ($\alpha_{asymp}$). The black dashed lines trace the actual peak incidence and the total number of fatalities in NSW during the simulated period.

#### 1.7.6 Summary

The local sensitivity analysis showed robustness of the model to changes in several key input parameters: SD intervention threshold, global transmission scalar, duration of infectious period, fraction of symptomatic cases in children, and asymptomatic infectivity. The highest sensitivity of the outputs variables (the peak incidence and the number of total fatalities) was detected with respect to changes in the infectious period's duration and the asymptomatic infectivity. Nevertheless, the model was shown to be robust in the neighbourhood of the default parameterisation, especially within the policy-relevant range of the compliance with stay-at-home orders, that is, $SD = 0.4$ to $SD = 0.5$.

## 1.8 Model Validation

The ABM was validated using the actual pandemic data in NSW during the period between 16 June 2021 and 27 October 2021[21,24]. While the numbers of COVID-19-induced deaths and incidence are accessible for the entire period, the vaccination statistics categorised into doses and age groups for NSW are only available for the period since 1 July 2021. As a result, in our simulation, we assume that the NSW vaccination coverage was unchanged from 16 June 2021 to 1 July 2021 with the initial coverage mapped to 1 July 2021. The simulation of daily vaccinations starts on 2 July 2021 according to the profile matching Fig. 2. The other parameters for the COVID-19 transmission model are kept at their calibrated values, described in Section 1.6.

The incidence and the daily fatalities are plotted in Fig. 9 and Fig. 10, contrasting the actual time series and the profiles simulated with different SD interventions ($SD = 0.3$ to $SD = 0.6$), each triggered by a threshold of 400 cumulative cases. As mentioned earlier, the range of stay-at-home compliance between $SD = 0.4$ and $SD = 0.6$ concurs with the retrospective analysis of the NSW outbreak[2] and is supported by the actual mobility reduction data [25]. Figures 9 and 10 show that the closest match to the actual incidence data is given by $SD = 0.4$ and $SD = 0.5$, while the new fatalities align best with $SD = 0.5$ and $SD = 0.6$. These results validate the ABM, enabling its use in the scenarios aimed to derive and optimise adaptive SD interventions.

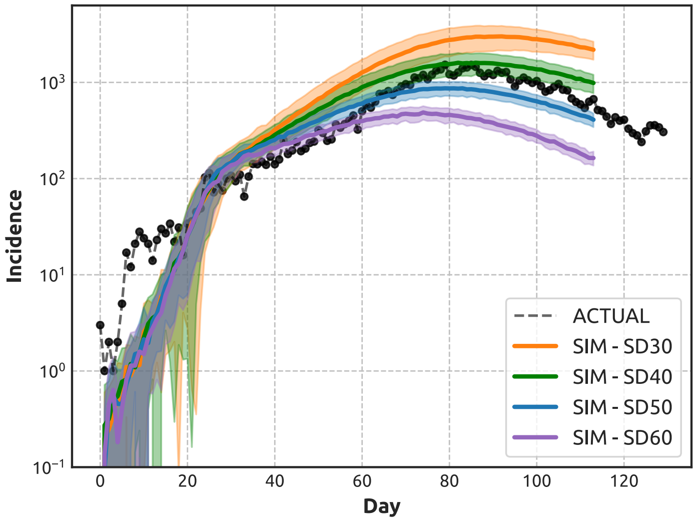


**Figure 9.** Model validation using actual COVID-19 incidence data in NSW, Australia, from 16 June 2021 (Day 0) to 12 October 2021 (Day 118), shown in log scale. The actual time series, shown in black, is obtained from NSW Health datasets[21]. The mean values and confidence intervals of simulations are shown in colour, varying across different levels of compliance with social distancing, from 30% to 60% (over 20 runs per scenario).

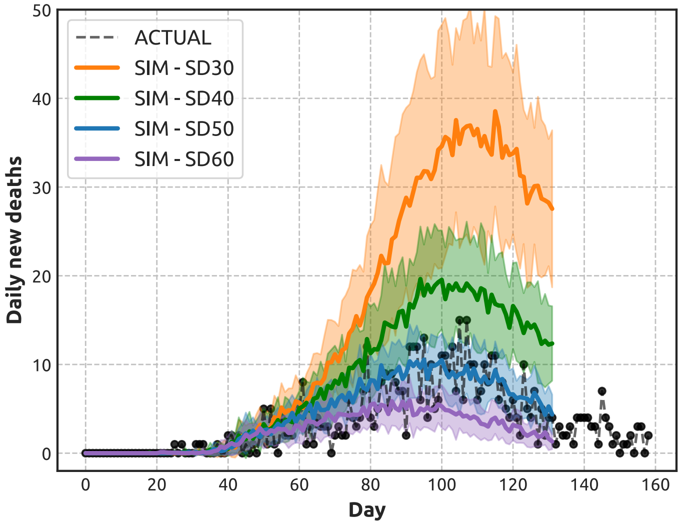


**Figure 10.** Model validation using actual COVID-19 data on daily fatalities in NSW, Australia, from 16 June 2021 (Day 0) to 12 October 2021 (Day 118). The actual time series, shown in black, is obtained from NSW Health datasets[24]. The mean values and confidence intervals of simulations are shown in colour, varying across different levels of compliance with social distancing, from 30% to 60% (over 20 runs per scenario).

## 2 Reinforcement Learning Approach to Search for Cost-Effective Social Distancing Policies

### 2.1 Period-wide NHB Objective Function

This section provides details for the setup of the period-wide net health benefit (PW-NHB) objective function used in Section 4.3. In order to evaluate the cost-effectiveness of an SD intervention policy, the NHB is calculated for the entire simulation period:

$$\begin{aligned} \text{PW-NHB} &= \mu_{E_{SD}} - \frac{\mu_{C_{SD}}}{\lambda} \\ &= \mathop{\mathbb{E}}_{\substack{a_t \sim \pi_\theta(o_t,\cdot) \\ (s_t,a_t,s_{t+1})\sim\tau \\ (s_t^0,a^0,s_{t+1}^0)\sim\tau^0}} \sum_{t=0}^{N} L(s_t^0,a^0) - \sum_{t=0}^{N} L(s_t,a_t) - \frac{\sum_{t=0}^{N} C(s_t,a_t) - \sum_{t=0}^{N} C(s_t^0,a^0)}{\lambda} \end{aligned} \tag{11}$$

where $\mu_{E_{SD}}$ and $\mu_{C_{SD}}$ are the mean values of measurements of the health effect and the cost of SD intervention during the entire simulation period; $\pi_\theta$ is the policy shaped by parameters $\theta$; action $a_t$ is sampled from policy $\pi_\theta(o_t,\cdot)$ based on the environmental observation $o_t$; the transition from $s_t$ to $s_{t+1}$ belongs to the trajectory $\tau$ controlled by SD interventions $a_t$; the transition from $s_t^0$ to $s_{t+1}^0$ belongs to the uncontrolled trajectory $\tau^0$ shaped by null action $a^0$; $L(s,a)$ is the health losses, measured in DALYs, resulting when the intervention at the SD level $a$ is applied to the environment at state $s$; $C(s,a)$ is the cost incurred between two consecutive time steps when an SD intervention $a$ is applied together with the base NPIs, i.e. CI and HQ, at the state $s$ of the environment; and $\lambda$ is the willingness-to-pay (WTP) parameter.

As mentioned in Section 4.1, the cost of SD intervention is estimated in proportion to the number of compliant agents:

$$\begin{aligned} \sum_{t=0}^{N} C(s_t,a_t) - \sum_{t=0}^{N} C(s_t^0,a^0) &\approx \sum_{t=0}^{N} \left[ C(s_t,a^0) + f(a_t)\,[C(s_t,a^1) - C(s_t,a^0)] - C(s_t^0,a^0) \right] \\ &\approx \sum_{t=0}^{N} \left[ f(a_t)\,[C(s_t,a^1) - C(s_t,a^0)] \right] \\ &\approx \sum_{t=0}^{N} f(a_t)\, C^1 \end{aligned} \tag{12}$$

where $f(a_t)$ is the SD compliance level associated with the action $a_t$, $a^0$ is the action for zero SD intervention, and $a^1$ is the action for full 100% SD intervention. In general, the baseline costs for CI and HQ, i.e., $C(s_t,a^0)$ and $C(s_t^0,a^0)$, may vary according to the number of incident cases. However, in this study, for simplicity, we assumed their values to be constant at every time step. In addition, we also assumed that the mean value $C^1$ of the costs for the full 100% SD intervention, i.e. $C(s_t,a^1) - C(s_t,a^0)$, is also known. These assumptions contribute to the approximations taken in Eq. 12. Hence, the objective function expressed by Eq. 11 is reduced as follows:

$$\text{PW-NHB} \approx \mathop{\mathbb{E}}_{\substack{a_t \sim \pi_\theta(o_t,\cdot) \\ (s_t,a_t,s_{t+1})\sim\tau \\ (s_t^0,a^0,s_{t+1}^0)\sim\tau^0}} \sum_{t=0}^{N} \left[ L(s_t^0,a^0) - L(s_t,a_t) - \frac{f(a_t)\,C^1}{\lambda} \right] \tag{13}$$

### 2.2 Empirical Convergence in the Training of SD Policies

Figures 11-13 show convergence of the training phase under different combination of WTPs ($10,000/DALY, $50,000/DALY, and $100,000/DALY) and $SD_{max}$ (30%, 50%, and 70%).

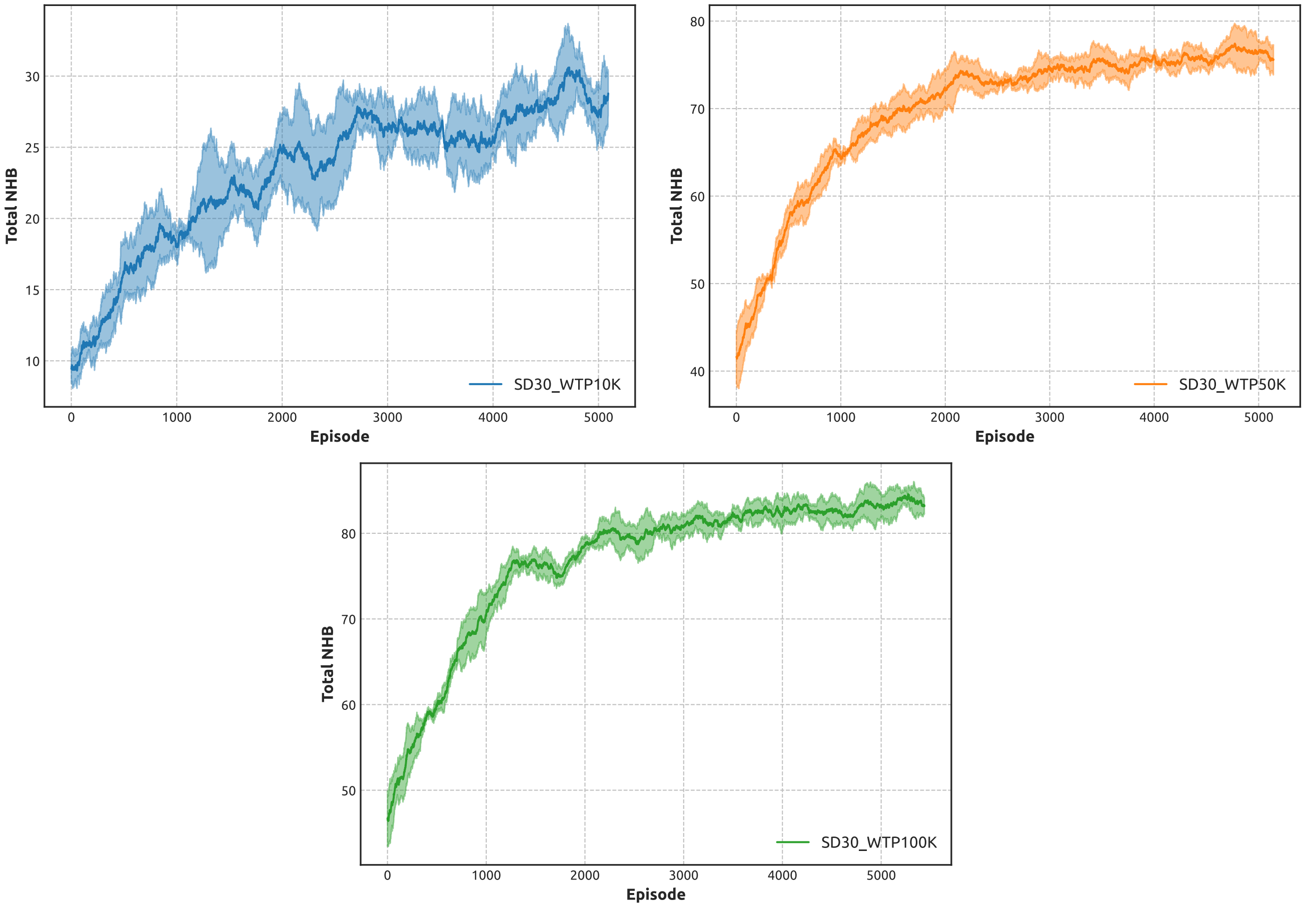


**Figure 11.** Accumulated episodic rewards generated over training episodes by the SD interventions trained under different combinations of WTP and $SD_{max} = 0.3$. Solid curves represent the mean values and the shaded areas represent standard deviation.

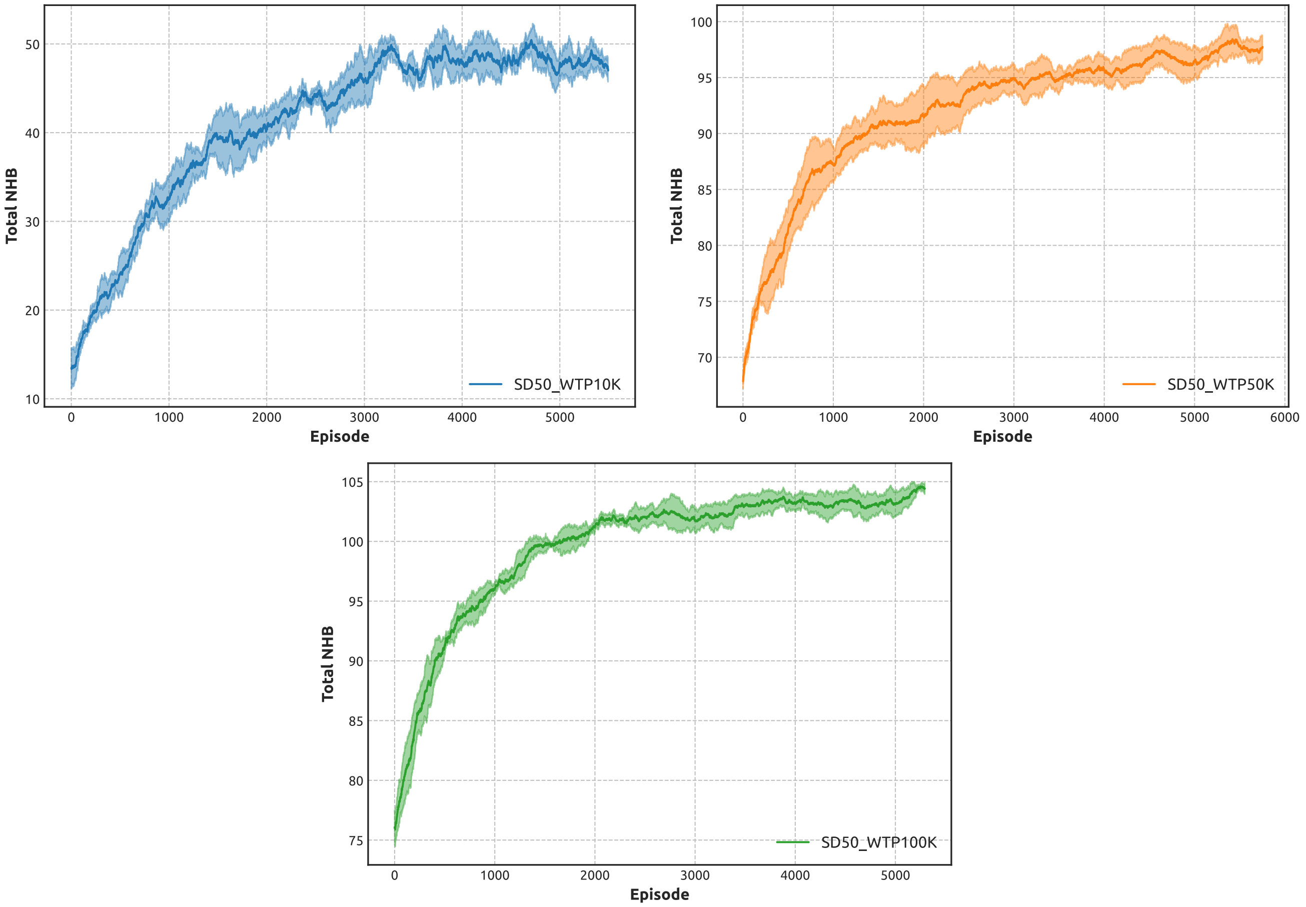


**Figure 12.** Accumulated episodic rewards generated over training episodes by the SD interventions trained under different combinations of WTP and $SD_{max} = 0.5$. Solid curves represent the mean values and the shaded areas represent standard deviation.

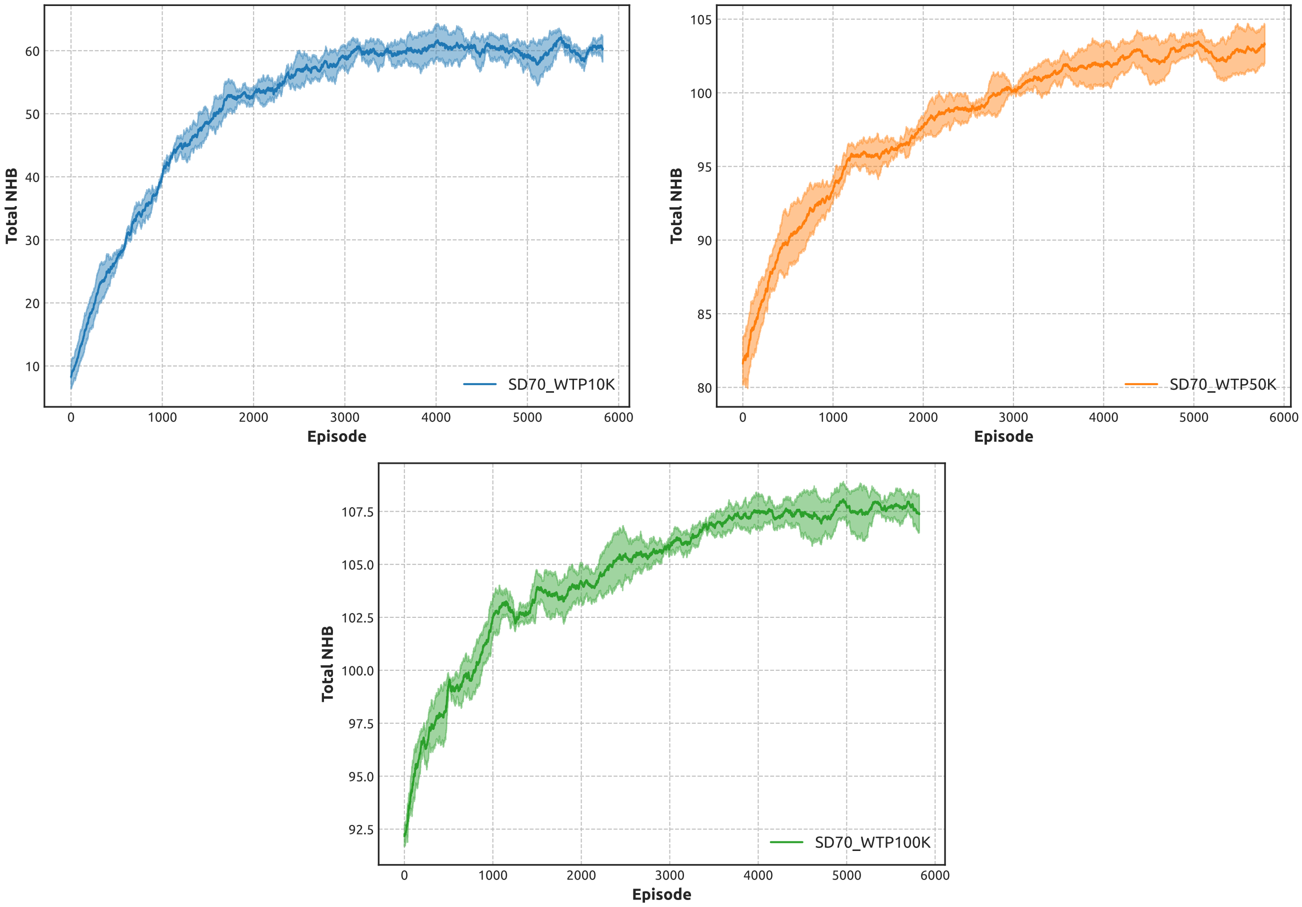


**Figure 13.** Accumulated episodic rewards generated over training episodes by the SD interventions trained under different combinations of WTP and $SD_{max} = 0.7$. Solid curves represent the mean values and the shaded areas represent standard deviation.

## 2.3 PPO Algorithm

Proximal Policy Optimization (PPO) algorithm was developed by[26] as a policy gradient actor-critic method which approximates both policy and value function. The architecture includes two components. The first component is the actor neural network used to make decisions about actions. The second component is the critic neural network used to predict the value function which is defined as the expected accumulated reward that the decision-maker can receive since a state. In our PPO implementation, the value function $V(s)$ is approximated by the critic network based on the observation of the decision-maker when the state of the environment is $s$. The critic neural network can be trained using the Temporal Difference (TD) Learning with the loss function specified by $L_t^{Vf} = \delta_t^2$, for

$$\delta_t = r_{t+1} + \gamma V(s_{t+1}) - V(s_t) \tag{14}$$

where $r_{t+1}$ is the reward received at the time step $t+1$, $\gamma$ is the discount factor, and $s_t$ and $s_{t+1}$ are the states of the environment at the time steps $t$ and $t+1$ along the trajectory $\tau$ controlled by the SD intervention.

The objective function used to optimise the actor is a clipped “surrogate" objective function defined as follows:

$$J(\theta_k) = L^{CLIP}(\theta_k) = \hat{\mathbb{E}}_t \left[\min\left(\phi_t(\theta_k)\hat{A}_t, \mathrm{clip}\left(\phi_t(\theta_k), 1-\varepsilon, 1+\varepsilon\right)\hat{A}_t\right)\right] \tag{15}$$

where $\hat{\mathbb{E}}_t$ denotes the empirical expectation over a batch of samples at the time step $t$, index $k$ is the current learning step, $\theta$ is the set of the parameters determining the actor, $\phi_t(\theta_k) = \frac{\pi_{\theta_k}(a_t|s_t)}{\pi_{\theta_{k-1}}(a_t|s_t)}$ is the ratio of the probabilities of taking action $a_t$ according to the stochastic policies parameterised by $\theta_k$ and $\theta_{k-1}$, with $\theta_{k-1}$ being the parameters of the previous policy used before the update, $\varepsilon$ is a hyperparameter that limits the range of $\phi_t(\theta_k)$ to $[1-\varepsilon, 1+\varepsilon]$ within the *clip* operation. Finally, $\hat{A}_t$ is the estimation for the advantage function at the time step $t$ which is computed as follows:

$$\hat{A}_t = \delta_t + (\gamma\upsilon)\delta_{t+1} + \ldots + (\gamma\upsilon)^{T-t+1}\delta_{T-1} \tag{16}$$

where $\gamma$ is the discount factor, and $\upsilon$ is the weight discount used in the generalised advantage estimation by the policy gradient implementation. In our study, we applied the standard version of PPO algorithm[26] implemented by[27] with the default set of hyperparameters.

### 2.4 Extended Results

In this study, we modelled adaptive SD interventions, optimised for their cost-effectiveness across different settings of maximal compliance with social distancing ($SD_{max}$) and "willingness to pay" (WTP). Section 2 presented our results and analysis contrasting two $SD_{max}$ levels, 30% and 70%, across all three considered WTP levels ( \$10K per DALY, \$50K per DALY, and \$100K per DALY). In this section, we include additional figures, comparing the medium and high $SD_{max}$ settings: 50% and 70%. As before, the training and simulations for each value of $SD_{max}$ were repeated for all three WTP levels. The dynamics of the optimised adaptive NPIs (Fig. 14), the resultant dynamics of net health benefit (Fig. 15), and the associated NHB components including the economic costs and health effects (Fig. 16) are presented to complement figures shown in Section 2.

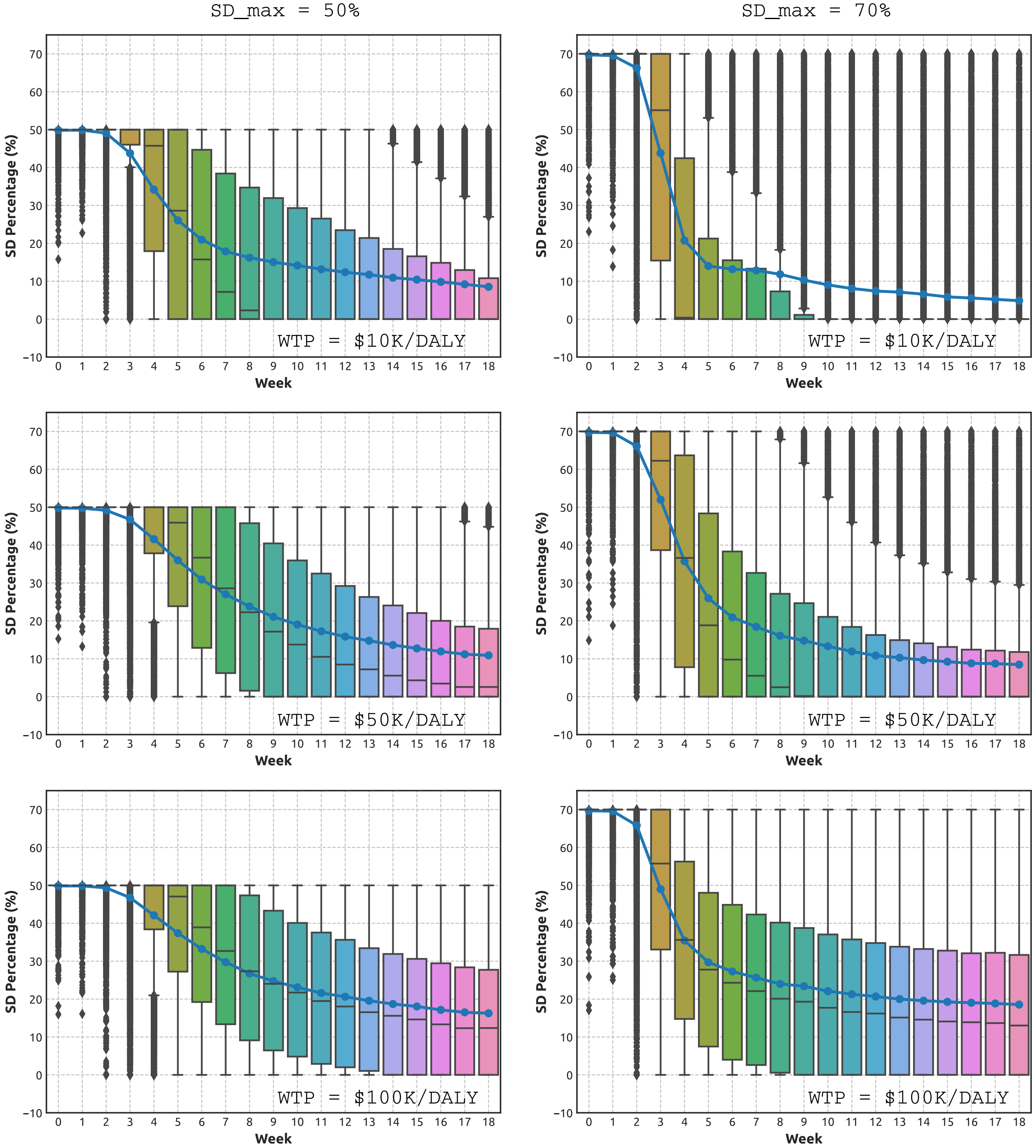


**Figure 14.** Adaptive NPIs, learned under different combinations of maximal SD levels $SD_{max}$ and WTP, over more than 14,000 simulations. Left: $SD_{max} = 0.5$. Right: $SD_{max} = 0.7$. Top: WTP is set at $10K per DALY. Middle: WTP is set at $50K per DALY. Bottom: WTP is set at $100K per DALY. Boxplots show the distribution of data over the quartiles, with box body capturing the mid-50% of the distribution. The curves shown with blue colour trace the mean values of the SD levels attained in each week. Outliers are shown in black.

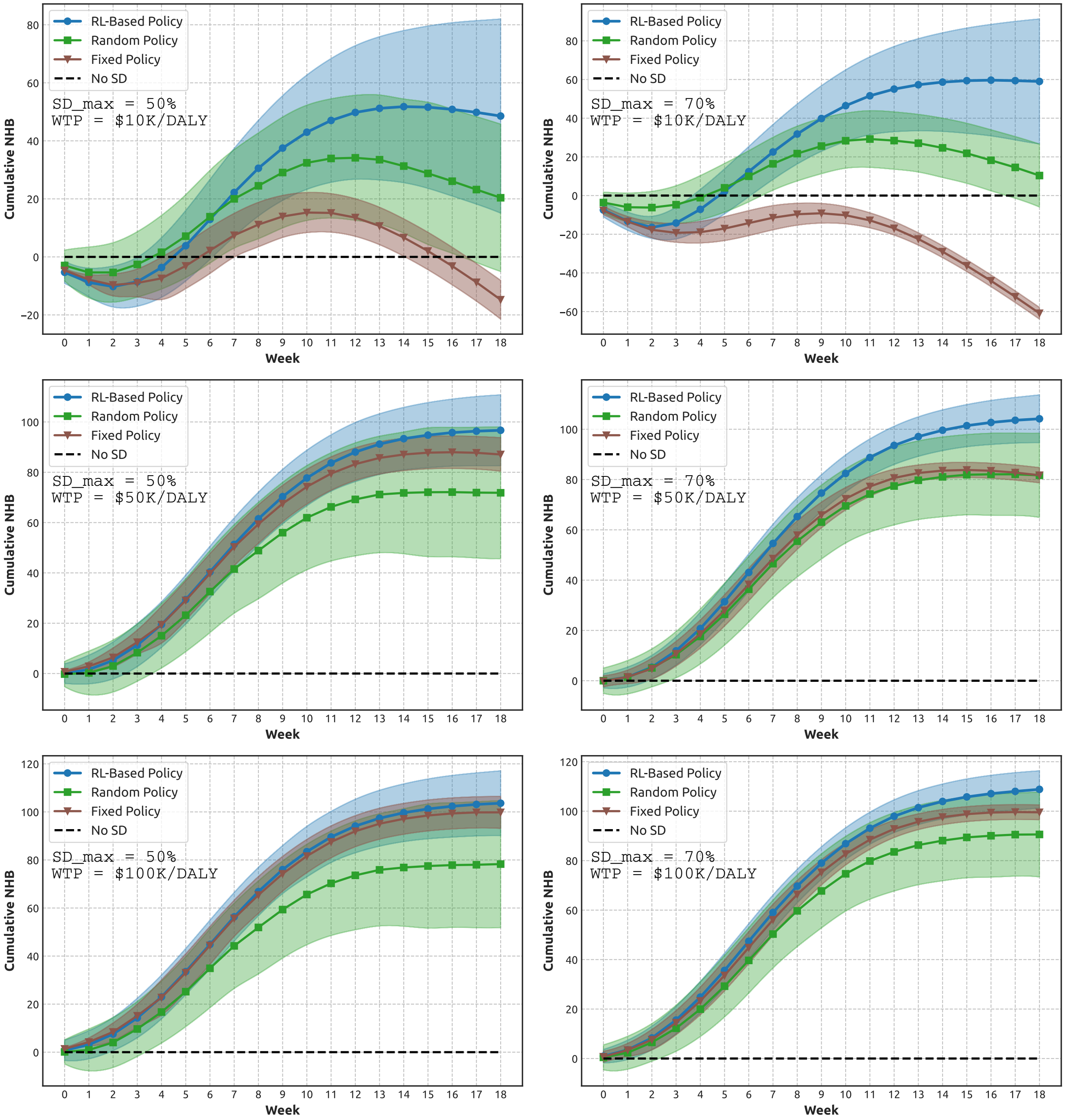


**Figure 15.** A comparison of cumulative Net health benefit (NHB) generated by the adaptive NPIs, fixed SD NPIs, random SD policies, and zero SD policies. Left: $SD_{max} = 0.5$. Right: $SD_{max} = 0.7$. Top: WTP is set at $10K per DALY. Middle: WTP is set at $50K per DALY. Bottom: WTP is set at $100K per DALY. Shaded areas show standard deviation.

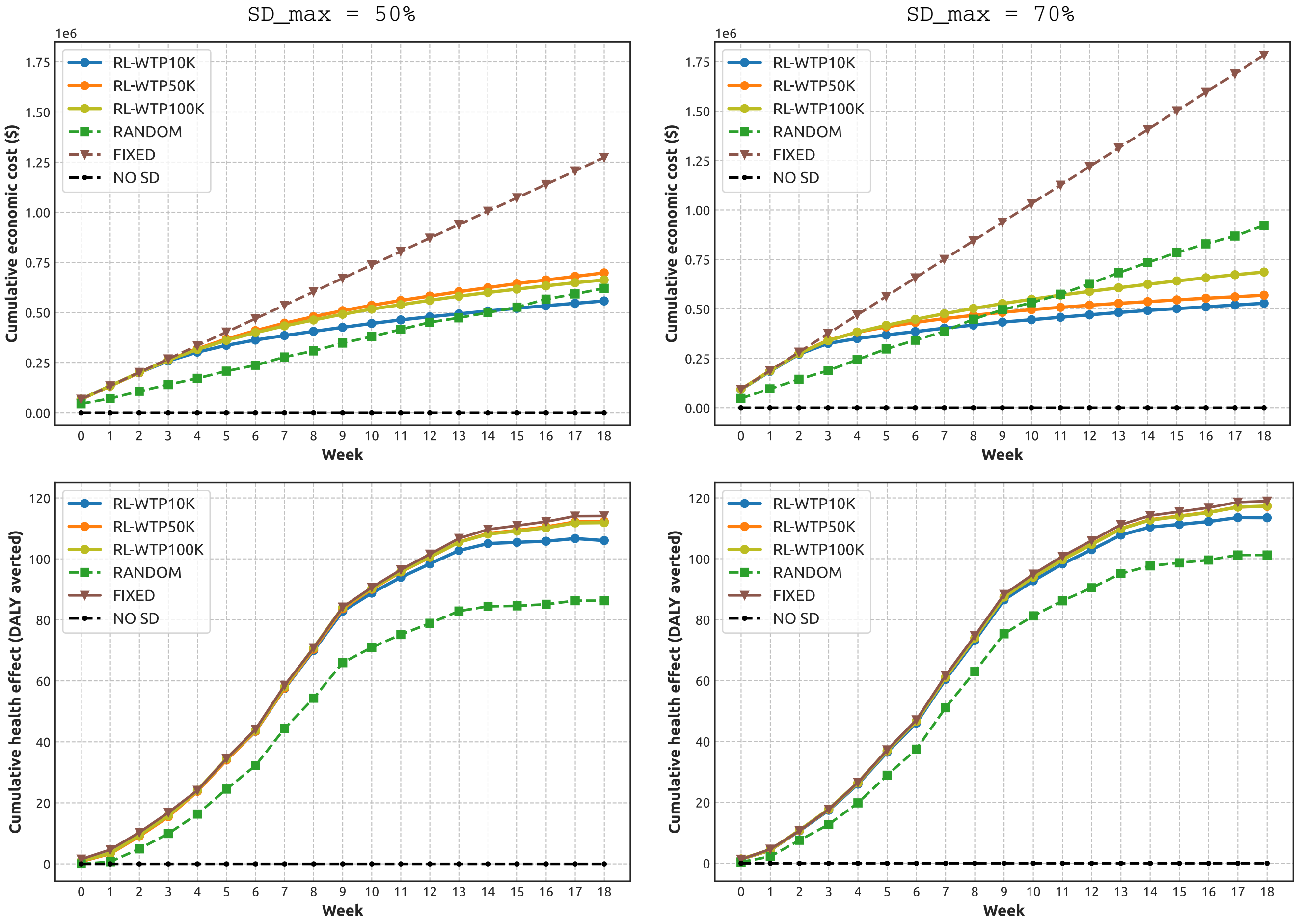


**Figure 16.** Components of the Net Health Benefit (NHB): mean values of cumulative economic costs (dollars) and cumulative health effect (DALY averted), shown for different NPIs: adaptive SD with three WTP thresholds ($10K per DALY, $50K per DALY and $100K per DALY), random SD, fixed SD, and zero SD. Left: the maximal SD level $SD_{max}$ is set at 50%. Right: the maximal SD level $SD_{max}$ is set at 70%.

## References


1. Chang, S. L., Harding, N., Zachreson, C., Cliff, O. M. & Prokopenko, M. Modelling transmission and control of the COVID-19 pandemic in Australia. *Nat. Commun.* **11**, 5710, DOI: 10.1038/s41467-020-19393-6 (2020).
2. Chang, S. L., Cliff, O. M., Zachreson, C. & Prokopenko, M. Simulating transmission scenarios of the Delta variant of SARS-CoV-2 in Australia. *Front. Public Heal.* **10**, 823043, DOI: 10.3389/fpubh.2022.823043 (2022).
3. Levin, A. T. *et al.* Assessing the age specificity of infection fatality rates for covid-19: systematic review, meta-analysis, and public policy implications. *Eur. J. Epidemiol.* **35**, 1123–1138, DOI: 10.1007/s10654-020-00698-1 (2020).
4. Fisman, D. N. & Tuite, A. R. Progressive increase in virulence of novel sars-cov-2 variants in ontario, canada. *medRxiv* DOI: 10.1101/2021.07.05.21260050 (2021). https://www.medrxiv.org/content/early/2021/08/04/2021.07.05.21260050.full.pdf.
5. Verity, R. *et al.* Estimates of the severity of coronavirus disease 2019: a model-based analysis. *The Lancet Infect. Dis.* **20**, 669–677, DOI: 10.1016/S1473-3099(20)30243-7 (2020).
6. Department of Health, Australian Government. COVID-19 Vaccine Roll-out (01/07/2021-27/10/2021) (2021). Accessed from July 1, 2021, to October 27, 2021.
7. Therapeutic Goods Administration. COVID-19 vaccine weekly safety report - 16-09-2021 (2021).
8. Zachreson, C., Chang, S. L., Cliff, O. M. & Prokopenko, M. How will mass-vaccination change COVID-19 lockdown requirements in Australia? *The Lancet Reg. Heal. - West. Pac.* **14**, 100224, DOI: 10.1016/j.lanwpc.2021.100224 (2021).

9. Lopez Bernal, J. *et al.* Effectiveness of Covid-19 Vaccines against the B.1.617.2 (Delta) Variant. *New Engl. J. Medicine* **385**, 585–594, DOI: 10.1056/NEJMoa2108891 (2021).

10. Harris, R. J. *et al.* Effect of vaccination on household transmission of SARS-CoV-2 in England. *New Engl. J. Medicine* **385**, 759–760, DOI: 10.1056/nejmc2107717 (2021).

11. Scientific Advisory Group for Emergencies. LSHTM: Interim roadmap assessment – prior to Step 4, 9 June 2021 (2021).

12. Campbell, F. *et al.* Increased transmissibility and global spread of SARS-CoV-2 variants of concern as at June 2021. *Eurosurveillance* **26**, DOI: 10.2807/1560-7917.ES.2021.26.24.2100509 (2021).

13. Agency for Clinical Innovation. Living Evidence - SARS-CoV-2 variants (2022).

14. Liu, Y. & Rocklöv, J. The reproductive number of the Delta variant of SARS-CoV-2 is far higher compared to the ancestral SARS-CoV-2 virus. *J. Travel. Medicine* **28**, taab124, DOI: 10.1093/jtm/taab124 (2021).

15. Diekmann, O., Heesterbeek, J. A. & Metz, J. A. On the definition and the computation of the basic reproduction ratio R0 in models for infectious diseases in heterogeneous populations. *J. Math. Biol.* **28**, 365–382, DOI: 10.1007/BF00178324 (1990).

16. Germann, T. C., Kadau, K., Longini, I. M. & Macken, C. A. Mitigation strategies for pandemic influenza in the United States. *Proc. Natl. Acad. Sci.* **103**, 5935–5940 (2006).

17. Zachreson, C., Fair, K. M., Harding, N. & Prokopenko, M. Interfering with influenza: nonlinear coupling of reactive and static mitigation strategies. *J. The Royal Soc. Interface* **17**, 20190728 (2020).

18. Liguoro, I. *et al.* SARS-COV-2 infection in children and newborns: a systematic review. *Eur. J. Pediatr.* **179**, 1029–1046, DOI: 10.1007/s00431-020-03684-7 (2020).

19. Davies, N. G. *et al.* Age-dependent effects in the transmission and control of COVID-19 epidemics. *Nat. Medicine* **26**, 1205–1211, DOI: 10.1038/s41591-020-0962-9 (2020).

20. National Centre for Immunisation Research and Surveillance, N. COVID-19 in schools and early childhood education and care services – the experience in NSW: 16 June to 31 July 2021. Tech. Rep. (2021).

21. NSW Data. COVID-19 cases by notification date, location...

22. ABC News. Tighter restrictions for Greater Sydney as NSW records 44 new COVID-19 cases. (2021).

23. Macartney, K. *et al.* Transmission of SARS-CoV-2 in Australian educational settings: a prospective cohort study. *The Lancet Child & Adolesc. Heal.* **4**, 807–816, DOI: 10.1016/S2352-4642(20)30251-0 (2020).

24. COVID-19 Data - Australia. COVID-19 deaths in Australia.

25. COVID-19 Data - Australia. Mobility data in NSW LGAs | COVID-19 latest data.

26. Schulman, J., Wolski, F., Dhariwal, P., Radford, A. & Klimov, O. Proximal Policy Optimization Algorithms. *arXiv:1707.06347 [cs]* (2017). ArXiv: 1707.06347.

27. Raffin, A. *et al.* Stable-baselines3: Reliable reinforcement learning implementations. *J. Mach. Learn. Res.* **22**, 1–8 (2021).
